# Perceived displacement explains wolfpack effect

**DOI:** 10.3389/fpsyg.2014.01423

**Published:** 2014-12-16

**Authors:** Matúš Šimkovic, Birgit Träuble

**Affiliations:** Department Psychologie, Universität zu KölnCologne, Germany

**Keywords:** representational momentum, perception of animacy, intention, agency, goal-directed behavior, chasing, position judgment

## Abstract

We investigate the influence of perceived displacement of moving agent-like stimuli on the performance in dynamic interactive tasks. In order to reliably measure perceived displacement we utilize multiple tasks with different task demands. The perceived center of an agent's body is displaced in the direction in which the agent is facing and this perceived displacement is larger than the theoretical position of the center of mass would predict. Furthermore, the displacement in the explicit judgment is dissociated from the displacement obtained by the implicit measures. By manipulating the location of the pivot point, we show that it is not necessary to postulate orientation as an additional cue utilized by perception, as has been suggested by earlier studies. These studies showed that the agent's orientation influences the detection of chasing motion and the detection-related performance in interactive tasks. This influence has been labeled wolfpack effect. In one of the demonstrations of the wolfpack effect participants control a green circle on a display with a computer mouse. It has been shown that participants avoid display areas with agents pointing toward the green circle. Participants do so in favor of areas where the agents point in the direction perpendicular to the circle. We show that this avoidance behavior arises because the agent's pivot point selected by the earlier studies is different from where people locate the center of agent's body. As a consequence, the nominal rotation confounds rotation and translation. We show that the avoidance behavior disappears once the pivot point is set to the center of agent's body.

## 1. Introduction

Recently, Gao and Scholl demonstrated (Gao et al., 2009, 2010; Gao and Scholl, 2011) that certain movement cues influence the detection of chasing motion and the performance in interactive tasks. These tasks usually present an array of moving sprites which we will collectively refer to as *agents*. The participant controls one additional sprite (chasee) with a computer mouse and tries to flee the agents. In Gao et al. (2009) the authors focused on cues that describe different types of goal-directed motion. In Gao et al. (2010) the authors focused on chasers' orientation. The orientation cue was defined as a rotation without translation. By adjusting the rotation while keeping the position/trajectory of the agents constant, the authors were able to study orientation independent of other motion cues. They found that in visual scenarios where multiple agents were oriented toward a common chasee, this impeded participant's performance as compared to control conditions where the agents' orientation pointed 90 degrees relative to the chasee's position. For example, in their Experiment 2^1^, the participants controlled a green circle with a computer mouse and tried to avoid contact with a white circle which chased the green circle. The display included six other randomly moving white circles which served as distractors and made the task difficult. As a manipulation, the authors added seven white darts that in half of the trials were oriented toward the green circle and in the other half perpendicular to it. They compared the two conditions in terms of the proportion of trials where the chaser caught the green circle. Even though the participants were explicitly told to ignore the darts, they were worse at escaping in trials where the darts pointed toward the green circle. In their Experiment 4^2^, the chaser was the only white circle in the display, so it was easily identified. However, the participants were additionally required to avoid contact with randomly moving darts. The escape rate was lower in trials where the darts were oriented toward the green circle in comparison to trials where the orientation was perpendicular. In Experiment 3a^3^, the comparison was not between trials, but rather the display was divided into areas which contained randomly moving darts with different orientations as explained in **Figure 3A** (in this report). The participants' task was to avoid contact with the darts. The authors found that in doing so, the participants spent more time in areas with darts oriented perpendicular to the green circle than in areas where the darts pointed directly toward it. The authors called the consistent negative influence of a head-on orientation on participant's performance *wolfpack effect*.

**Figure 1:**
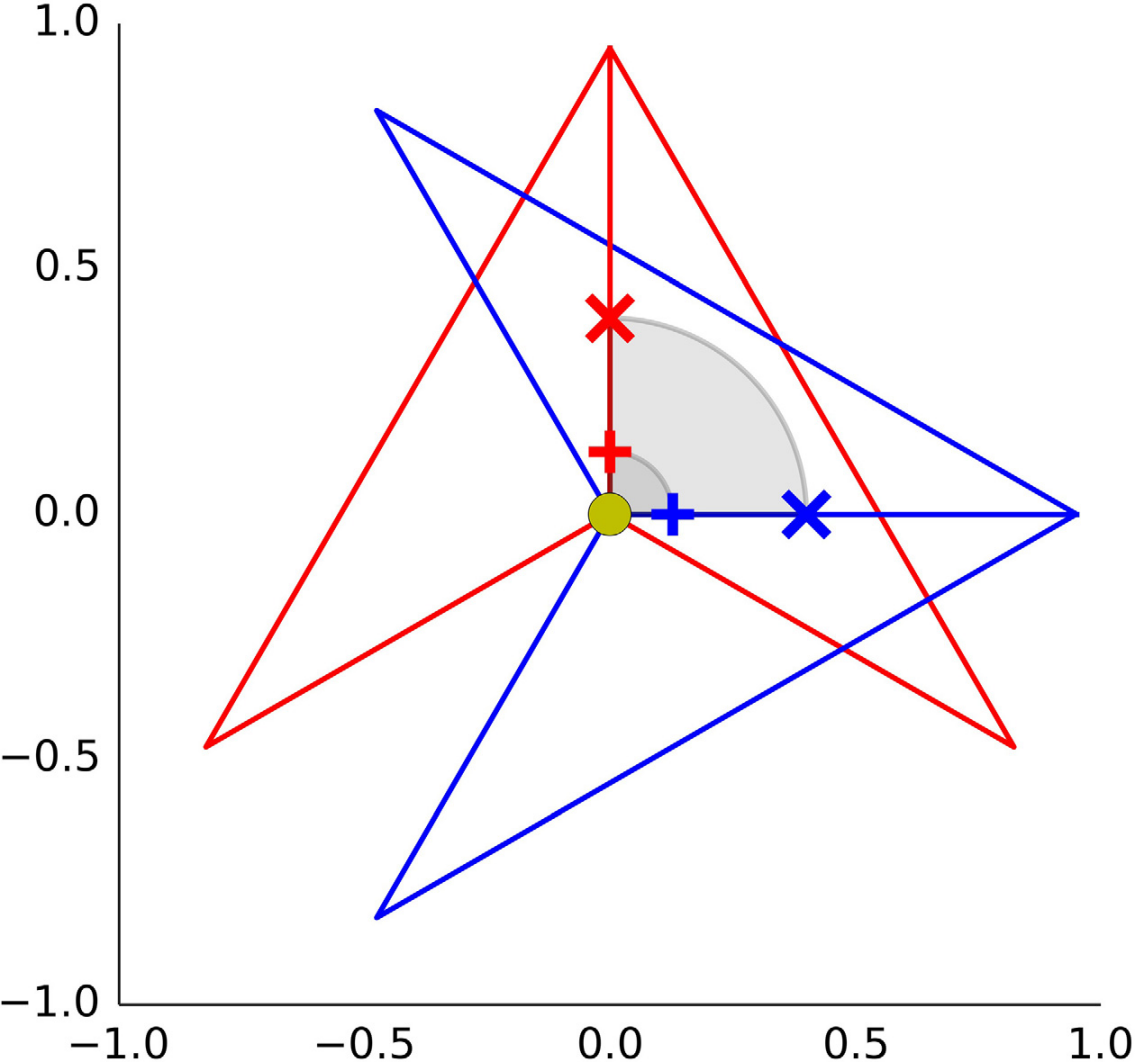
**The red dart is rotated around its pivot (yellow dot)**. If the perceived center of dart's body lies somewhere between the pivot and the dart's nose, the nominal rotation is perceived as translation. This is demonstrated with two points: the center of mass located 0.13 degrees away from the concave vertex (+), and a point at 0.4 (×).

If we want to conclude that the wolfpack effect is due to the agent's orientation, one assumption is crucial. The experimentally manipulated orientation change needs to be perceived as an orientation change and not as a translation of the center of agent's body. This is illustrated in Figure 1 on the example of the darts used by Gao et al. (2010). We need to distinguish between the location of the pivot point, which is selected by the experimenter, and the center of agent's body which needs to be inferred from participant's behavior. To further stress this distinction we sometimes use labels *nominal* pivot and *perceived* center. The authors designated the concave vertex (yellow dot) as the nominal pivot. Intuitively however, we do not perceive the concave vertex as the center of agent's body. The center of mass (+) looks like a much better candidate. What happens if the perceived center is displaced away from the nominal pivot in the direction of the center of mass? In this case, the rotation around the pivot is perceived as a translation from the old position (red +) to a new position (blue +). If the agent rotates toward the chasee, this is perceived as a translation toward the chasee. Furthermore, the traveled distance is larger, the further away the perceived center is from the nominal pivot (compare × and +). Consequently, we can contrive an alternative explanation for the results in Gao et al. (2010). In Experiment 3a, the participants avoided the areas with darts oriented toward the chasee because they perceived these agents as moving toward the green circle. We assume that in Experiment 4, participants estimated the critical distance to the surrounding agents. Then, based on this estimate, they decided where to move the green circle next. If the agents' perceived location was shifted toward the chasee in wolfpack trials, the participants would often prematurely leave the current location in exchange for another alternative location. This choice may be reasonable with respect to the perceived position, but would be a bad choice from the point of view of the dart's pivot point (yellow dot). It was the distance from the nominal pivot that was used to score whether the green circle was caught or not. As a consequence, in wolfpack trials the participants would be penalized for their misperception of the agent's center while with perpendicular darts there would be no penalty. The results of Experiments 3a and 4 can be alternatively interpreted in terms of domain general processes, such as distance estimation and decision making. Under our alternative explanation, the orientation does not play any role and the wolfpack effect would disappear if the pivot point would be set to where people set the center of agent's body.

Experiment 3b in Gao et al. (2010) was the only experiment that did not use darts. The design and results were similar to that of Experiment 3a, except that instead of darts the participant tried to avoid white circles whose orientation was determined by the direction of two red dots (“eyes,” see e.g., **Figure 7** for an example). We refer to these stimuli as bugs. With a bug the center of mass is identical to the pivot used in Gao et al. (2010)—it's the center of the bug's circular contour. Still, this does not mean that people do not perceive the center of the bug's body as shifted (e.g., toward the direction of its eyes).

The literature on memory displacement is relevant here (Hubbard, 2005). It has been repeatedly demonstrated that if participants are asked to reproduce the last position (i.e., the center of its body) of a stimulus that just disappeared on the screen, participant's responses are systematically displaced by factors such as gravity, momentum, or shape. Crucially, some studies [e.g., Freyd and Miller, 1992 as cited in Hubbard (2005)] demonstrated that displacement can also be influenced by information about the stimulus animacy. Other studies showed that in general the surface properties of the stimulus object influence the displacement (Reed and Vinson, 1996; Vinson and Reed, 2002). Memory displacement is studied by asking the participants to report the last position of an object that just disappeared. This is usually done by asking participants to select the last position with a computer mouse, (e.g., Hubbard and Bharucha, 1988) or alternatively, by asking them to decide whether a probe is presented at the same location where the object disappeared or at a different location (e.g., Freyd and Finke, 1984). The research on memory displacement showed that its magnitude can be independent of the objective physical principles (Hubbard, 1997) and may diverge from participant's explicit beliefs (Freyd and Jones, 1994). That is, even if the center of mass and the participant's explicit beliefs are consistent with the pivot used by Gao et al. (2010), nevertheless a memory displacement may occur.

But, how are such displacements in recall tasks relevant to the implicit interactive tasks used in Gao et al. (2010)? According to Hubbard's computational theory of memory displacement (Hubbard, 2005, p. 844), “displacement occurs because it aids in the spatial localization of physical objects and facilitates rapid motor responding to objects in the environment.” He further adds (cf.) that “accurate spatial localization is important for calibrating an observer's response to a stimulus so that a maximally effective and adaptive interaction with that stimulus might be achieved.” For the sake of illustration consider the phenomenon of representational momentum. The displacement due to the representational momentum anticipates the future position of a moving object. Such anticipation corrects the discrepancy between the object's position when the action is programmed and when the action is performed. This interpretation of the role of displacement is supported by studies that show that the displacement diminishes in less uncertain contexts where such anticipation is not needed/possible (e.g., representational momentum diminishes if the movement of the object is controlled by the participant Jordan and Knoblich, 2004).

We can apply the lessons from the literature on memory displacement to the stimuli and tasks in Gao et al. (2010) and their Experiment 3b in particular. In Experiment 3b, it is important for the participant to accurately predict the direction of the object's motion. This helps to avoid collision. Then we should expect a memory displacement which is influenced by factors that provide cues for the future motion. Since biological organisms usually move in the direction in which their bodies and eyes are oriented, agent's orientation is a good candidate for such a cue. Thus, it is plausible that the perceived center of the bugs is displaced in direction of its eyes and for similar reasons, the recalled center of the darts may be shifted even farther toward the nose than the center of mass would predict.

In the current study we investigate the influence of perceived displacement of the center of an agent's body on participant's performance in the interactive tasks from Gao et al. (2010). In particular we revisit the task from their Experiment 3, the so-called Leave-Me-Alone task (LMA). We choose this experiment since it is the only one that demonstrated the wolfpack effect with the bug stimuli. We use both the bugs and the darts, since these provide different cues to where the agent's center may be located (surface properties and shape, respectively).

Our argument can be distilled into two separate claims:

1. The perceived center of the dart and the bug stimuli is displaced away from the nominal pivot used by Gao et al. (2010) in the direction of the agent's nose/eyes.
2. This displacement influences participants' avoidance of wolfpack areas in the LMA task. In particular, the participants avoid wolfpack areas because the nominal rotation toward the chasee in the wolfpack areas is perceived as a translation toward the chasee.

**Figure 2:**
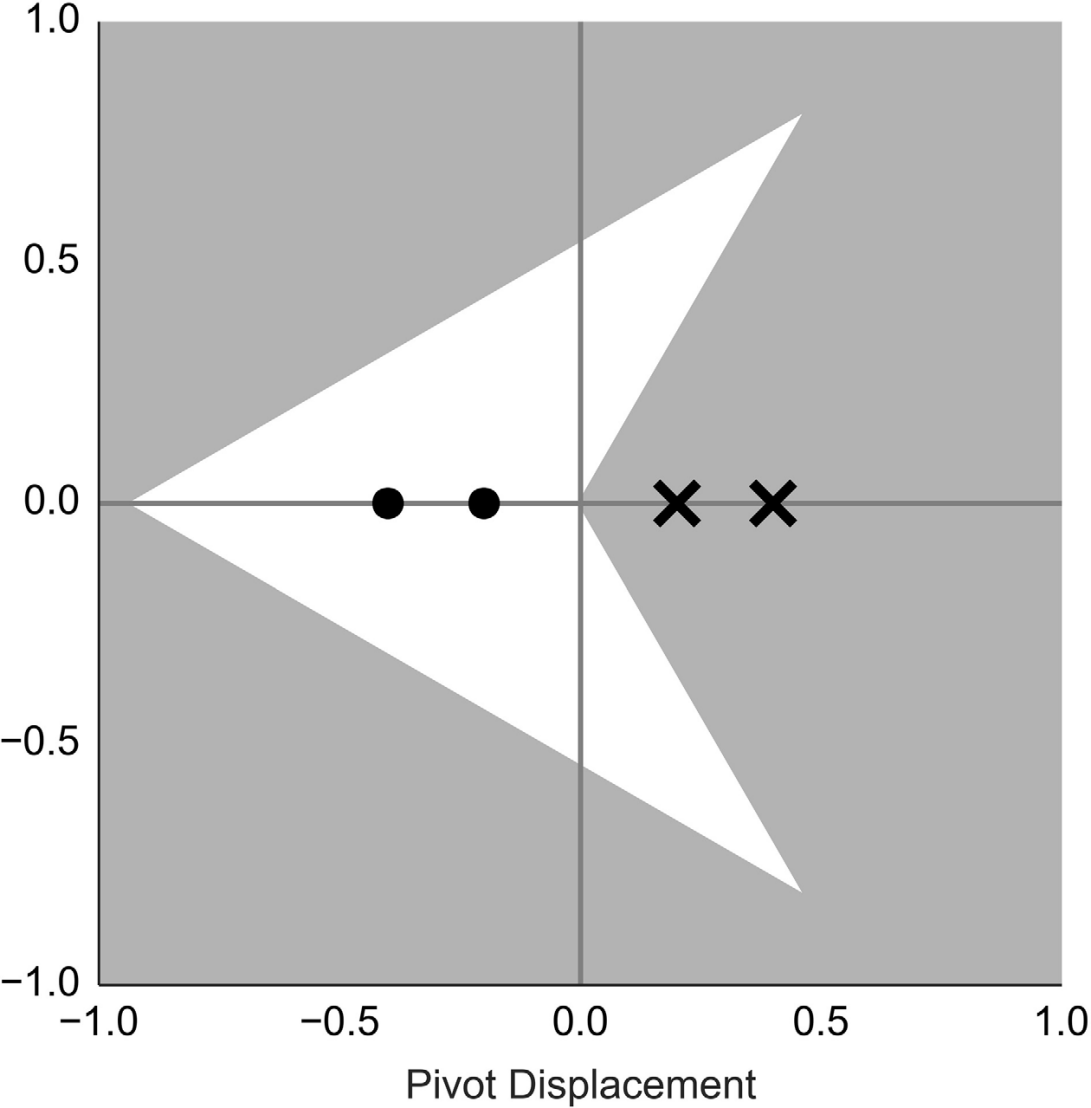
**Dots and crosses show where the pivot of the dart was set in Block 2 and Block 3**. The concave vertex [the pivot in Gao et al. (2010)] is chosen as a zero reference point. Negative values shift the pivot toward the nose, while positive values shift in the opposite direction. This choice of negative values reflects the following logic: If people perceive the agent's center to be shifted by *k* degrees in the direction of its nose, then we need to shift the position of the pivot by −*k* degrees, so that the pivot and the agent's center are alligned.

Consider the second claim first. To test the second claim we shift the pivot along the anteroposterior axis of the agent as explained in Figure 2. We measure how this manipulation influences participant's avoidance of wolfpack areas. We need to separate the constant influence of the orientation cues from the influence of our manipulation. With bugs, we can simply turn off the orientation cues by omitting the eyes, i.e., by using white circles as control stimuli. With darts, there is no straightforward way to neutralize the constant influence of the shape on the perceived displacement. Instead, we measure participants' avoidance behavior over a range of displacement values and use a regression model to separate the two factors in the analysis.

**Figure 3:**
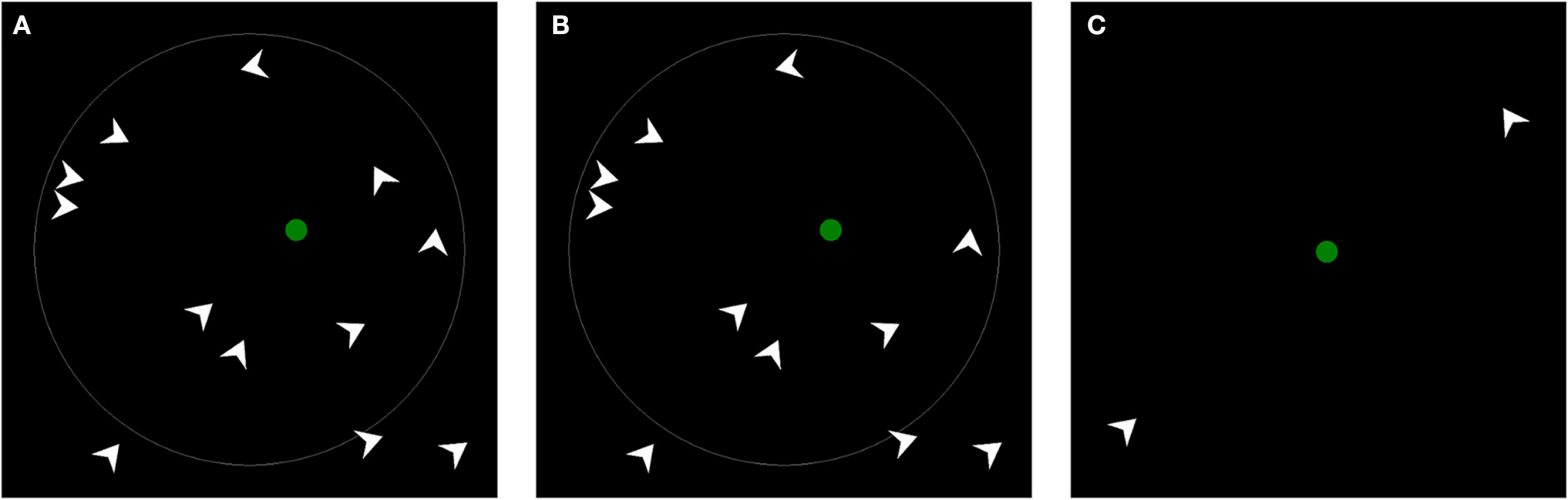
**Task overview. (A)** shows LMA task used by Gao et al. (2010) in experiment 3a. By horizontally and vertically bisecting the display into two halves we obtain four quadrants. Three agents move randomly inside of each quadrant. In two quadrants the agents are oriented toward the green circle (the two quadrants on the left) and in other two perpendicular to it. Agent's direction of motion is independent of its orientation. The participant's task is to avoid contact with agents. **(B)** Shows Location Recall task. After 17 s of the LMA task, the motion stops and one agent vanishes (in this case, the dart located to top-right from the green circle). The participant is asked to select with a cross-hair (not shown) the last position where he had seen the agent. **(C)** Shows Distance Bisection task. The participant is asked to move the green circle such that it stays equidistant to the two randomly moving agents. Note, the gray circle indicating the boundary for mouse movement may be difficult to see in print.

To test the first claim we utilize two tasks. First, we append a location recall task to the LMA task (Figure 3B). Immediately after each trial of the LMA task, one dart/bug disappears while all the remaining objects freeze at their last position (Figure 3B). The participant is asked to select with the computer mouse the position where the agent disappeared. Second, we devised a novel Distance Bisection task (Figure 3C). The participants are shown the same motion as in the LMA task. However, only two agents from two different quadrants are displayed. One agent is oriented toward the chasee while the other one is oriented perpendicular to it. The participants are asked to move a green circle so that it stays on the shortest path between the two agents and equidistant to both. This is done while the two agents are moving. The task thus requires the participant to constantly move the green circle. We expect that the movement of the green circle will be slightly displaced in the direction away from the agent, pointing at the circle and toward the perpendicular circle. Since the Distance Bisection task measures displacement without engaging memory, we label the phenomenon perceived displacement or sometimes just displacement. The adjective perceived should indicate that the participant's judgment and not some veridical feature of the environment (e.g., agent's center of mass) is the locus of the displacement.

The use of multiple tasks with different task demands allows us to identify the perceived displacement more reliably. Participants are not oblivious to the displacement in their judgments and may reflect and partially compensate the discrepancies (Courtney and Hubbard, 2008). The Distance Bisection task avoids these pitfalls. The constantly changing motion pattern of the two agents engages the participants in a dynamic real-time behavior. There is little opportunity to monitor, reflect, and correct the judgment. Furthermore, one may object that an observed displacement in the Location Recall task is due to a post-perceptual shift in the memory, but the LMA task does only remotely engage memory. The Distance Bisection task is not susceptible to this objection.

On the other hand, the Location Recall task is presented immediately after each LMA trial and in this respect, is more directly connected to the LMA task. Furthermore, the Location Recall task allows us to connect our findings to the literature on memory displacement. Finally, we add few trials that measure memory displacement of the static stimuli.

So far, we have framed our study as an attempt to investigate the influence of a potential confound in a previously published study. However, the current study is also of interest to the researchers studying memory displacement and its role in action. If our claims are correct, our study should provide another demonstration in which the action context is relevant to the perceived displacement. The import of our study is our use of multiple tasks with different task demands and multiple stimuli with different properties. This allows us to better identify the factors influencing the displacement than studies that focus on a single measure.

## 2. Methods

### 2.1. Participants

Forty one psychology students (seven male) participated in the study in exchange for a course credit. All participants had normal or corrected to normal vision. The average age was 21.7 years. The age ranged from 18 to 33 years.

### 2.2. Stimuli

The stimulus programming followed the description in Gao et al. (2010) and the examples provided on the web-page of Brian Scholl who is a co-author of the Gao et al. (2010) study. The display was divided into four quadrants, each forming a square of 5.9 degrees. Three white objects moved inside each quadrant. These objects were either bugs or darts. A bug was a circle (1.9 degrees in diameter) that had two red eyes drawn on the top. The eyes were two red circles of size 0.19 degrees, located 0.71 degrees from the center of the white circle and 0.49 degrees apart. The dart was a white polygon with one concave and three convex vertices as shown in Figure 2. The convex vertices were arranged on a equilateral triangle with the concave vertex at its center. The distance from each convex vertex to the center was 0.95 degrees. The participant steered a green circle (chasee, 1.2 degrees diameter) with the computer mouse. The movement was confined to lie within a circular area of 11.75 degrees radius. The boundary of the circular area was shown on the screen as a thin gray line. The motion of the agents was generated as follows. The agents moved at a constant speed of 7.8 degrees per second. Each object changed its direction at random intervals with 3 direction changes per second on average. The new direction was chosen randomly from a uniform range of −45 to 45 degrees around its current direction. The objects were pervasive to each other. Upon touching the wall of the designated quadrant, they bounced off—the movement trajectory was mirrored, while the orientation remained unaffected. As in Gao et al. (2010), when the green circle overlapped with a white agent, the surface of the agent turned red. After testing several participants with bugs, we got the impression that the red color did not motivate the participants enough. We therefore added a beep tone which sounded whenever a collision happened, and as long as the two shapes overlapped (see Section Unsuccessful Replication below for more details).

The orientation was manipulated as follows. The agents were oriented either with their eyes/nose directed toward the agent's location, or perpendicular to the agent. There were two quadrants with perpendicular oriented agents and two quadrants with head-on oriented agents (wolfpack). The layout of the quadrants was randomly chosen on each trial.

### 2.3. Tasks and design

We tested the bug and the dart stimuli in two separate sessions with different samples of participants. Each experimental session consisted of 3 blocks with 42 trials per block. The experiment with the static stimuli consisted of 13 trials. These were appended to the 42 trials of the last block. An overview of all conditions and the amount of obtained data is shown in Supplementary Table 1. For some participants not all blocks were run. This was because the fixed time we had alloted for the session has been exceeded. We now provide a detailed description.

#### 2.3.1. Block 1

At the start of each trial the circular boundary and the initial position of the green circle was displayed. The participant initiated the trial by bringing a cross-hair mouse cursor to the green circle and by clicking on its surface. Furthermore, we ensured that the circle's initial position was at least 4 degrees away from the nearest agent. Then, the agents appeared and the LMA task started (Figure 3A). The participant tried to avoid contact with the agents by moving the green circle inside the circular boundary. This lasted 17 s. We appended a location judgment task at the end of the trial. After the movement ended, the agents and the chasee remained stationary. One agent disappeared (Figure 3B). The participant then clicked on the last location where he saw the missing agent with a cross-hair mouse cursor. The missing agent was chosen randomly from the three agents nearest to the chasee. Whenever possible, the computer program selected an agent that did not overlap with others and such that the perpendicular and the wolfpack agents were chosen approximately equally often. As in Gao et al. (2010), in our first block the pivot was at the concave vertex.

#### 2.3.2. Block 2

In the second block we used the same motion trajectories as in block 1, but the sprite was shifted along the anteroposterior axis away from the pivot point. Since we are concerned with the relative distance between the sprite and its pivot we will choose the concave vertex of the dart and the circle's center of a bug as an arbitrary reference point. Then the manipulation can be described by a relative distance of the pivot point to the reference point. This is illustrated in Figure 2. For the same reason we also allow ourselves to speak of “pivot shift” or “pivot displacement,” even though the pivot is set to the same position across all conditions. Strictly, it's the sprite that is shifted. However, if we wished to follow the exact nomenclature, we would need to introduce three terms: the pivot, the sprite location with respect to the pivot and the perceived location of the center of agent's body with respect to the sprite. This would complicate our exposition considerably.

We used multiple values of pivot displacement distributed equiproportionally and randomly across the block. We adjusted the set of pivot displacement values as the data became available. We wanted to select a set of pivot displacement values that were similar in magnitude to the perceived displacement of the agent's center (from the reference point) that we expected to find in the Location Recall task and in the Distance Bisection task. We tested white circles (these were identical to the bugs in shape but the two red eyes were omitted) with the first batch of participants. We started with a wide displacement set of {−0.15, −0.05, 0.05, 0.1, 0.15, 0.2}. Thus, the block consisted of seven trials of each type presented in a completely randomized order. Later we shifted the set to {0.05, 0.1, 0.15, 0.2 0.25, 0.3}. With the darts we first used values {−0.4, −0.2} and later {0.4, 0.2}. Otherwise, the trials in block 2 were identical to block 1. The displacement was either perpendicular or toward the green circle. The agents were organized into four quadrants based on the type of the displacement. Finally, each trial was followed by a Location Recall task. We included the Location Recall task in order to make the first two blocks as similar as possible. Furthermore, the Location Recall task allowed us to check whether the pivot displacement manipulation was successful.

#### 2.3.3. Block 3

In this block only two agents from two different quadrants were shown: one with perpendicular and one with head-on orientation. The initial position of the green circle was set at the nominal midpoint between the two agents. By nominal midpoint we mean the midpoint of the shortest path between the pivot points of the two agents. As in the previous blocks, each trial of the Distance Bisection task (Figure 3C) started after the participant clicked on the green circle. The participants were asked to move the green circle such that it stayed at the midpoint between the two agents. The first 18 trials were analogous to block 1 in that there was zero pivot displacement (plus the circles had eyes). In the remaining 24 trials the sprite was displaced in the direction of its orientation (and we showed circles instead of bugs). The schedule for the magnitude of pivot displacement was identical to the one used in block 2.

The last block concluded with 13 trials. These were meant to query participant's explicit understanding of where the agent's center is located. A static agent was shown for a random interval of 2–3 s. Then the cross-hair appeared and the agent either disappeared (in the first five trials) or it remained visible. The participants were asked to select the position of the agent with the mouse. In the experiments with the bugs, the eyes were displayed on the first 10 trials and their orientation was chosen randomly. Similarly, the orientation of the darts was chosen randomly.

### 2.4. Eyetracking

Immediately before taking part in the current experiment, the participants participated as an adult control group in an infant eye-tracking study. This experiment took 5–10 min. Since the participants were already seated at a calibrated eye tracker, we decided to include eye tracking measurements although no analyses of the eye tracking data were planned and none were performed.

### 2.5. Materials and data

The ethics committee of the Faculty of Behavioral and Cultural Studies at the Universität of Heidelberg approved the current study. The participants gave informed consent to participate in the study and were further given a written option to make their data publicly available. All participants agreed. The materials and the data are available from http://github.com/simkovic/wolfpackRevisited. Demonstration movies of the LMA task and the Distance Bisection task are available at http://vimeo.com/81181262 and http://vimeo.com/81181263, respectively.

### 2.6. Procedure

Participants were seated 50–70 cm away from the screen (all values in degrees of visual angle are based on 50 cm distance) of a Tobii T60 display with a built-in remote eye-tracker. The display was ran at 75 hz. In block 1 and block 3 the participants were given written instruction. In block 2 the participants were told that they will perform the same experiment as in block 1. An english translation of the instruction is provided in the repository. Upon the conclusion of the experiment, the participants were debriefed and dismissed. Each block lasted 15 min and there was a brief break between the blocks.

The experiment was presented and controlled with PsychoPy 1.77 (Peirce, 2007) and Tobii SDK 3.0.

### 2.7. Statistical modeling

In the analyses reported below we follow the approach advocated in Gelman and Hill (2007) and Gelman and Shalizi (2012). We first design a model that appropriately describes the data-generating process. We use posterior predictive checking to decide whether a model is acceptable. We start by fitting simple models and then increase their complexity. Usually, we start with a model with separate parameters for each participant. We then design a hierarchical model (Lee, 2011) that partially pools data across participants. Finally, we report and interpret the estimates of variables of interest—usually the estimates of the population parameters. We report the mean estimate and the 95% percentile interval of the estimate's distribution in the form *x*, 95% PI [*a, b*], where *x* is the mean estimate and *a* and *b* are the lower and upper bound, respectively.

The main advantage of this approach is that it gives us flexibility to design complex models that match (or at least approach) the complexity of the processes that generated the data. The model design involves decisions which may considerably alter the posterior estimates and even the conclusions of the analysis. We discuss some of these modeling decisions in the Results section below. In general, the main conclusions were supported across all the various models we evaluated, and can be considered robust.

An overview of the models from which we report the estimates is given in Supplementary Table 2. Hierarchical models are most easily formulated and evaluated within the bayesian framework. Bayesian models require the formulation of prior distributions for the analysis to proceed. We omitted these from the Supplementary Table 2. We selected priors such that they did not influence the estimation results. Usually we chose uniform priors, constrained to a reasonable range of parameter values (e.g., range between 0 and 1 for mean proportion μ_μ_ in S2.2). The models were evaluated with STAN 2.2.0^4^ which fits statistical models with Markov chain Monte Carlo. In each analysis, four chains were sampled and the convergence was checked by estimating the potential scale reduction $\hat{R}$ (see Gelman et al., 2003, p. 297) in the parameters. In all analyses and for all parameters $\hat{R}$ < 1.05 where $\hat{R}$ = 1 upon convergence. The analyses are documented and can be replicated with the IPython Notebooks available from the project repository. The Supplementary Table 3 helps to locate the reported results in these files.

## 3. Results

This section is structured as follows. We first report the results for each of our tasks separately and then report the between-task comparisons. The tasks are presented in the order of increasing complexity. We start with the static stimuli and move then to the Distance Bisection task. The Location Recall and the LMA task follow. We then analyze the mouse movement data from the LMA task. We close the results section with a displacement comparison across tasks and stimuli.

### 3.1. Localization and recall of static stimuli

**Figure 4:**
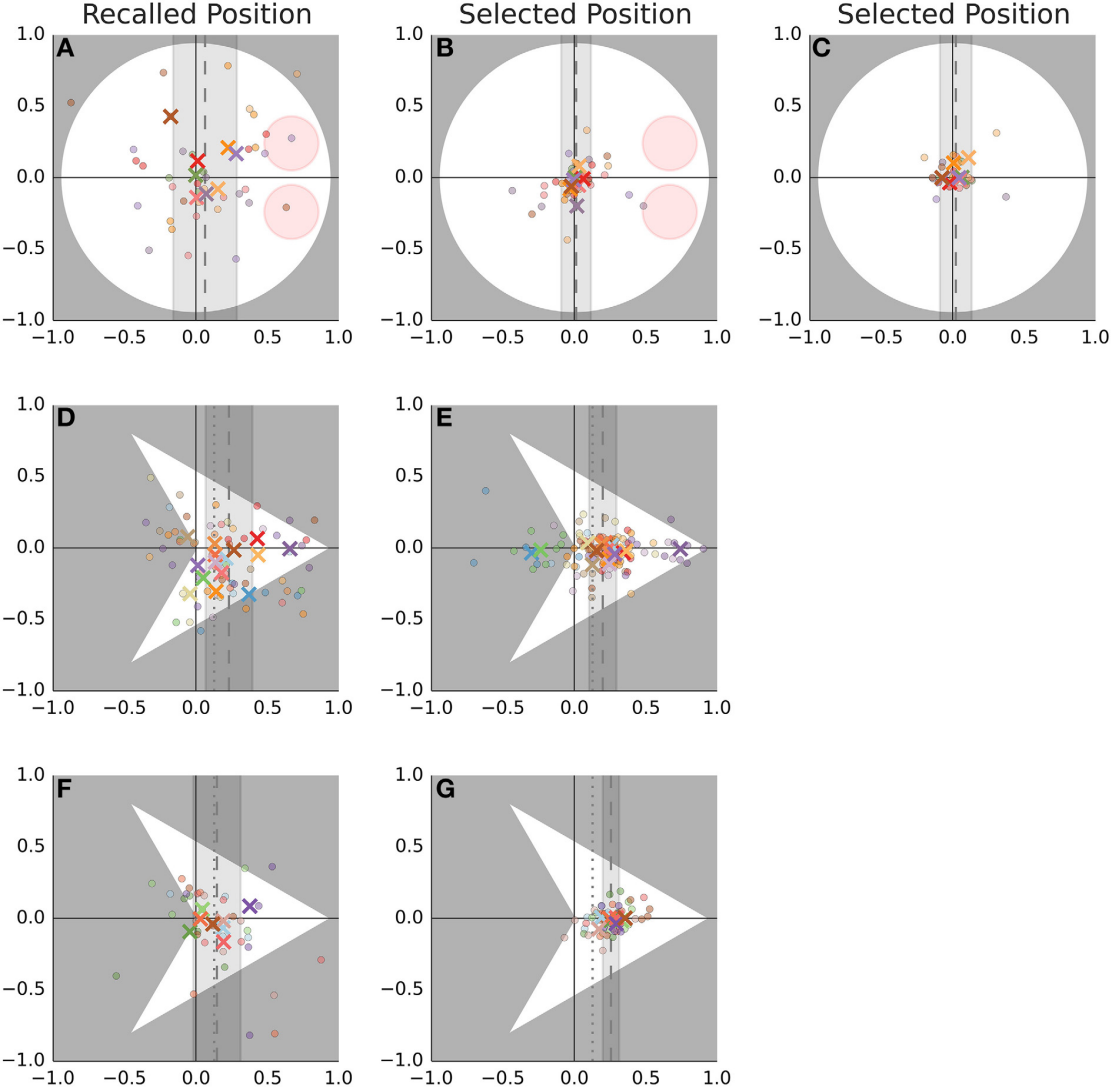
**Recall and localization of static stimuli**. The first row **(A–C)** shows the data obtained with bugs while the remaining rows show the data obtained with darts. As explained in the text, the latter sample was further split into two groups. This split is shown in the second and third row. The first column **(A,D,F)** shows the recalled position while the remaining columns show the positions selected while the stimulus remained visible. Each cell shows measurements that were rotated around the agents's nominal pivot from Gao et al. (2010) (this is the point [0, 0] in Figure 2) such that the direction of eyes/nose is aligned with the positive direction of the horizontal axis. Dots show individual trials while the crosses show the median for each participant. The color of the symbols distinguishes different participants and is consistent across columns. The agents are drawn to scale on the background of each cell. The dashed line indicates the mean estimate (across all participants and trials) of the displacement. The shaded area shows the 95% interval of the regression model. The dotted line in **(D–G)** shows the horizontal position of the dart's center of mass.

The measurements are shown in Figure 4 and summary statistics are given in Table 1. The localization judgments (B, C, E, G) are more precise than the recall performance (A, D, F). The darts show a systematic displacement in the direction of the nose (D–G). The bugs show no such displacement although the recall task (A) shows a small displacement that may be worth further investigation. The reported position is displaced further toward the nose than the center of mass (vertical dashed line at 0.13 in Figure 4) would predict.

**Table 1:** **Displacement of static agents**.

**Agent**	**Task**	**Figure cell**	**Mean displacement**
Bug	Recall	A	0.06 [−0.12, 0.25]
Bug	Localization	B	0.01 [−0.07, 0.01]
Circle	Localization	C	0.02 [−0.07, 0.11]
Dart	Recall	D and F	0.19 [0.12, 0.25]
Dart	Localization	E and G	0.22 [0.16, 0.27]

*The values in brackets give 95% CI computed with Student's *t* distribution (*df* = n_s_ n_v_ − *1*, R3.1 in Supplementary Table 3)*.

Recall that the task with the static stimuli was preceded by a task with stimuli where the pivot displacement was non-zero. Does the reported location of the static stimuli reflect learning and transfer of knowledge from the preceding task? The second row (D, E) shows data for the participants who saw the pivot shifted toward the nose (dots in Figure 2). Here, the data are consistent with a learning account. Participants put the agent's center where the pivot was located previously. However, in the third row (F, G) we would expect a judgment shifted toward the tail, but we don't observe any notable difference between the two groups of participants.

Finally, there are some striking differences between the participants. The dark purple participant located the center at the nose (E) and did so also during the recall (D). The green participant located the center behind the dart but only when the stimulus remained on the screen.

### 3.2. Distance bisection

#### 3.2.1. Bugs

**Figure 5:**
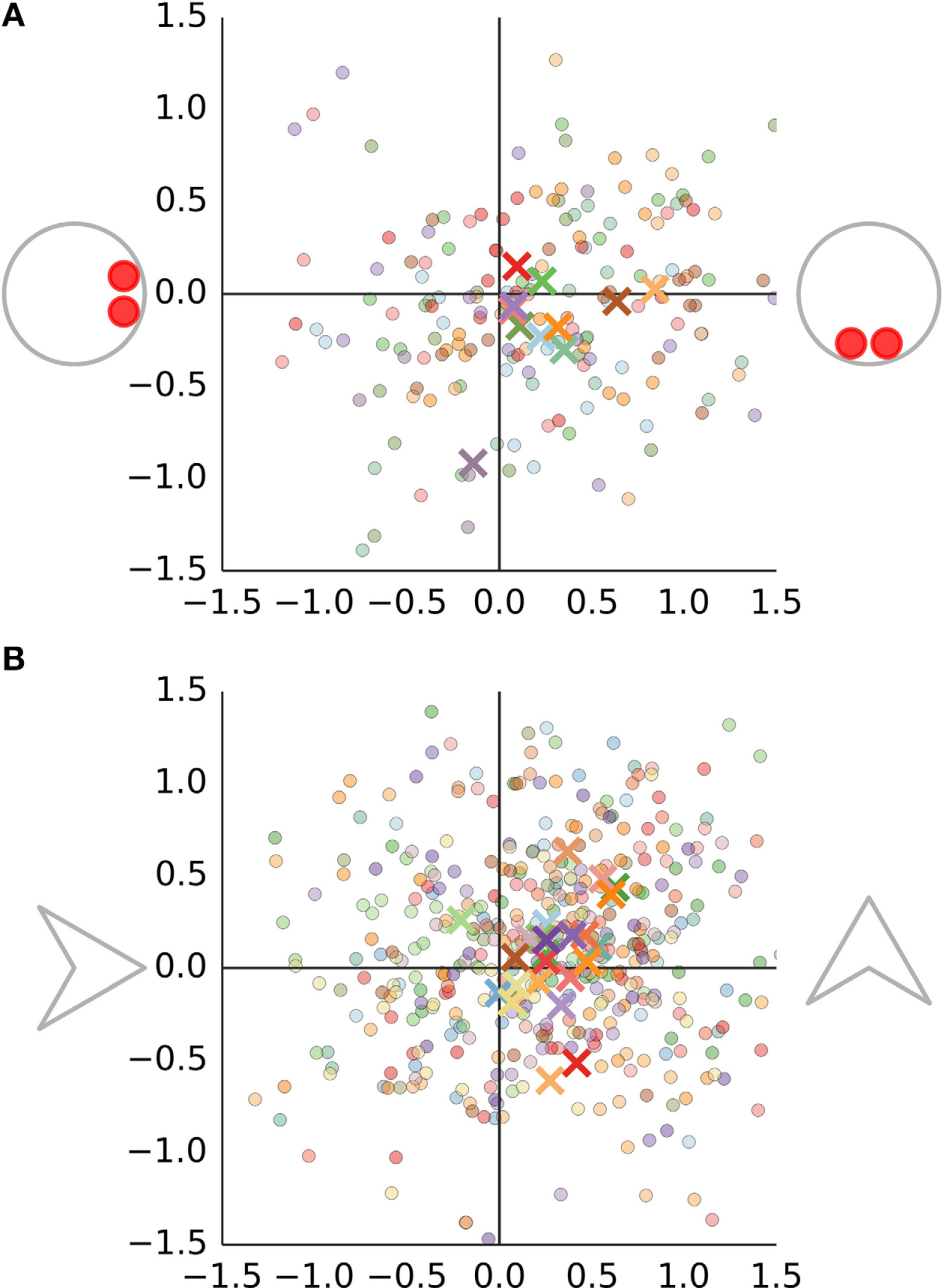
**The results of the Distance Bisection task for bugs are shown in (A) and for darts in (B)**. Each trial is shown as a dot. Each cross shows an average for particular participant. Data from different participants are identified by a different color.

Figure 5A shows the data from the trials where the bug's pivot was set to zero. Each trial took 17 s. The mouse position was sampled at 75 hz. We discarded the first 2 s and computed the average displacement of the green circle from the nominal midpoint. We rotated participants' judgments around the nominal midpoint such that the position of the head-on agent is aligned with the negative direction of the horizontal axis in Figure 5 and the perpendicular agent is located in the opposite direction. The nominal midpoint is located at [0, 0]. Since the participants would not only bisect the distance between the two agents, but also any shift due to the displacement of the agent's center which we wished to estimate, we multiplied the magnitude of the displacement on each trial on each axis by 2. As expected, most of the measurements and participant medians in Figure 5 are shifted toward bottom right. This is the direction of the combined influence of the displacement of the two agents. We use a hierarchical model (S3.1, R3.2 in Supplementary Table 3) to estimate the magnitude of the displacement on the horizontal and vertical axis of Figure 5 separately. We will refer to these as *head-on* and *perpendicular* displacement. (These are μ_μ,*x*_, μ_μ,*y*_ in S3.1.) Keep in mind that the head-on displacement describes the displacement due to the agent oriented toward the green circle while the perpendicular displacement is the displacement of the agent oriented perpendicular to it. Recall also that we always mean the displacement on the anteroposterior axis of the agent, as shown in Figure 2.

Direct displacement is 0.25, 95% PI [0.11, 0.39] degrees. The perpendicular displacement is 0.1, 95% PI [−0.02, 0.21] degrees. As can be seen in Figure 5, not all participants do show perpendicular displacement and as a result the estimate of the population mean is smaller.

#### 3.2.2. Darts

**Figure 6:**
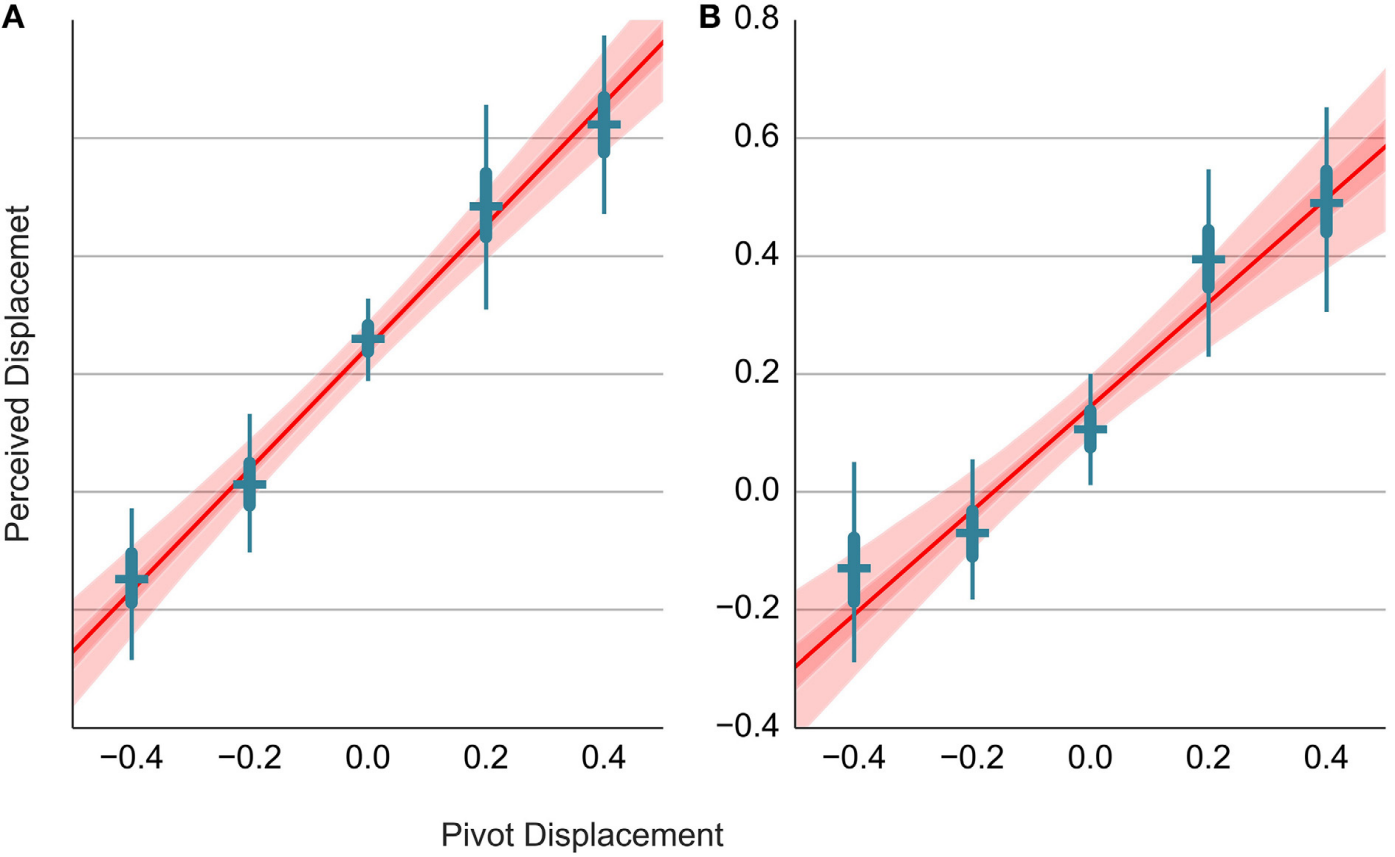
**Relationship between the pivot displacement and the perceived displacement in the Distance Bisection Task**. The head-on displacement is shown in **(A)**, while the perpendicular displacement is shown in **(B)**. The perceived displacement (vertical axis) is shown in relationship to the nominal pivot displacement (horizontal axis). Blue error bars show the estimates for the individual conditions. The results of fitting a regression line to the data from all conditions are shown in red. The red line shows the mean estimate, while the light and dark red band show the 95 and 50% interval, respectively.

The Bisection judgments for the dart stimuli (D2.3.1) are shown in panel B of Figure 5. Again, we fit a hierarchical model for displacement on each axis (R3.3). The same model is applied to the data from trials where the pivot was set to non-zero values (D2.3.2 and D2.3.3). The estimates of head-on displacement are shown as error bars in Figures 6A,B, respectively. The vertical axis shows the perceived displacement from the pivot. The estimates from different conditions in A lie on a line with a slope approximately equal to one. This would be expected since both the displacement of agent's center and the pivot location use the coordinate convention in Figure 2. We can use this fact to include all bisection data in a single regression model (R3.4) with the nominal pivot displacement as a predictor and the perceived displacement of the agent as a dependent variable. Consider the head-on displacement first. The fitted regression line is shown in red in Figure 6A. Its slope is 1.04, 95% PI [0.86, 1.21]. Thus, if we shift the dart by one degree in front of its pivot, this results in a perceived shift of the dart's center by 1.04 degrees and the participants move the green circle by 0.52 degrees away from the head-on agent. Of more interest is the offset of the regression line. The offset gives the perceived displacement across all conditions referenced at the dart's concave vertex and thus independent of the shift in the pivot. The offset is 0.25, 95% PI [0.2, 0.29] degrees. The displacement is non-zero and larger than the location of the center of mass at 0.13.

The results of the analogous analysis for the perpendicular displacement are shown in Figure 6B. The separate estimates for different pivot positions show that the data points do not lie on a line, but rather form a sigmoidal curve. If we fit a regression line to the data from different conditions, we observe a slope of 0.88, 95% PI [0.64, 1.12] and a constant offset of 0.15, 95% PI [0.09, 0.2].

#### 3.2.3. Circles

We fit the regression model (R3.5) to the bisection data which used circles with non-zero pivot displacement. In this case, there is no orientation cue since circles lack eyes. The slope of the head-on and perpendicular displacement is 1.06, 95% PI [0.26, 1.86] and 0.64, 95% PI [−0.17, 1.52], respectively. These estimates are very imprecise due to the small sample size. However, they indicate that the smaller slope of the vertical displacement is not a property of the dart stimuli but rather a general property of the task.

### 3.3. Location recall

#### 3.3.1. Statistical modeling

**Figure 7:**
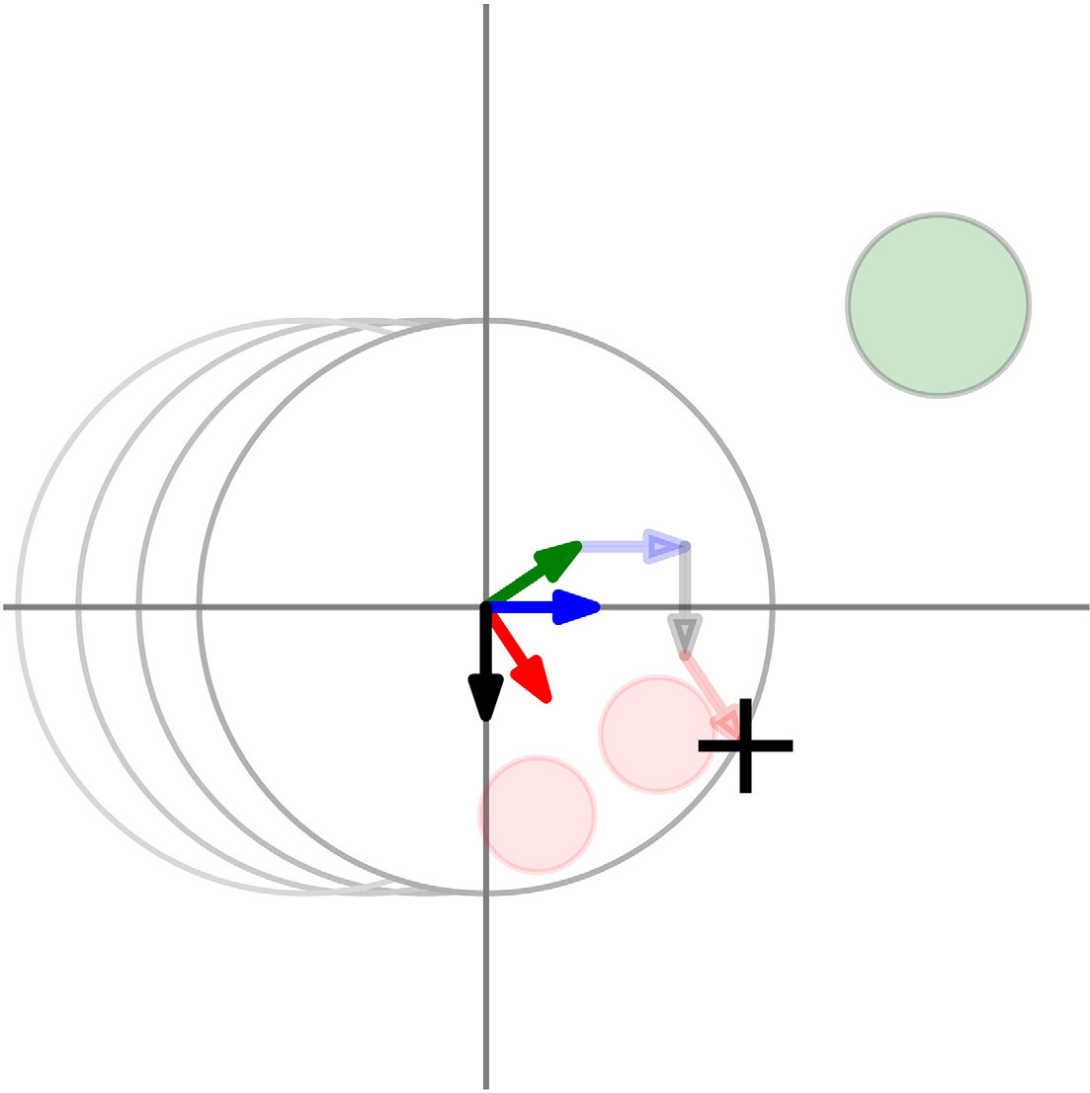
**Additivity of different displacement factors**. Four different factors influence the displacement (cross), namely representational gravity (black), representational momentum (blue) hand inertia (green) and agent's orientation (red). The total displacement due to these factors is a sum of the vectors (shown as the thick arrows at the origin). The vectors represent the direction and the magnitude of the different factors. One way to visualize the vector sum is to translate and superpose the individual vectors over each other, so that they form a connected path. This is shown by the transparent arrows.

With data from block 1 and 2, we evaluate the displacement of the agent's recalled location with respect to the position where the agent disappeared. We expect four factors to influence the displacement. These factors have been repeatedly reported in the literature on representational momentum. Their influence is illustrated in Figure 7. First, representational gravity pulls objects down along the vertical axis. Second, under the influence of a representational momentum participants extrapolate future positions along the object's direction of motion and recall these as the last position. Third, we expect that the position of the green circle (controlled by participant) will influence the judgment. In particular we expect that the recalled position will be pulled toward the last mouse position. Similar influence of the starting position on the judgment has been reported in line bisection tasks (Halligan and Marshall, 1989). We call this displacement factor the hand inertia. Finally, we call the displacement in the direction of the agent's orientation (as indicated by the direction of eyes/nose), orientation displacement.

**Figure 8:**
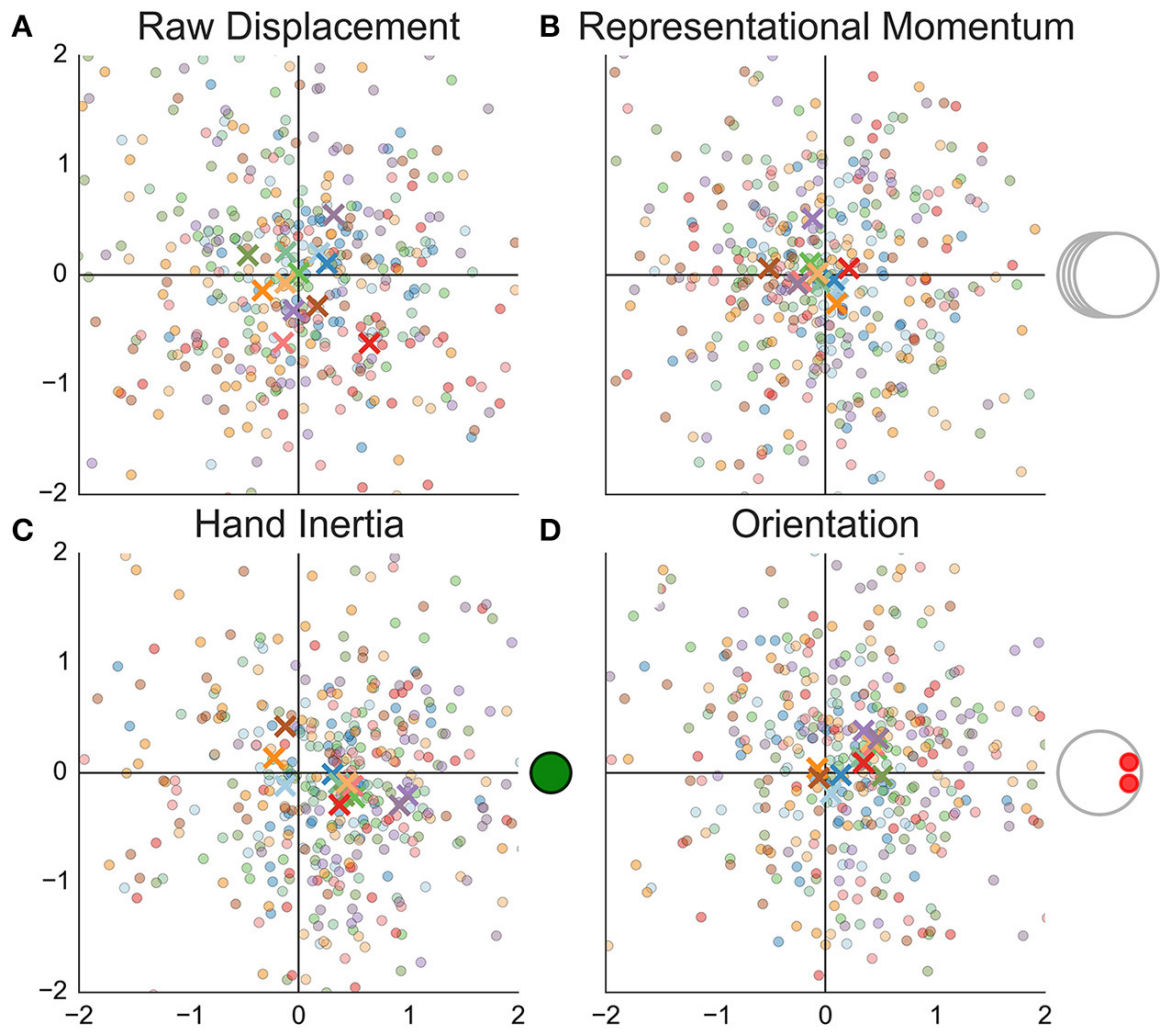
**Displacement in the Location Recall task**. Each of the four factors is shown in a separate panel. Panels **A–D** show respectively the raw displacement, representational momentum, hand inertia and orientation displacement. Dots in each panel show individual trials while the crosses show participant averages. Each participant is shown in a different color. The measurements are rotated, such that the orientation of the displacement factor across trials is aligned with the positive direction of the horizontal axis. Note, there are measurements beyond −2 and 2 degrees that are not shown. However, judgments with a distance to the reference point larger than 4 degrees were discarded from the analysis.

Some of the above mentioned factors are correlated due to the structure of our task. To disentangle their influence, we formulate a regression model. We assume that the influence of the four factors is linearly additive. This is illustrated in Figure 7. Vector addition has been proposed as mechanism by Hubbard (1995) to explain the interaction of multiple displacement factors. In support, Motes et al. (2008) have shown with principal component analysis that displacement due to gravity and due to momentum load on separate components. In our case, additivity is further made plausible by the pattern of results presented in Figures 8C,D. Furthermore, we assume a bivariate Gaussian distribution for errors. Again, the data in Figure 8 show that this assumption is plausible.

More formally, let **h** = [*h_x_, h_y_*] be the vector of the difference between the recalled position and the position (of the reference point) of the missing agent. Let ϕ_*k*_ be the angle given by the direction of the predictor *k* (in the respective order as listed in the previous paragraph). Then we are interested in the estimates of the regression coefficients α_*k*_ given by the equation 
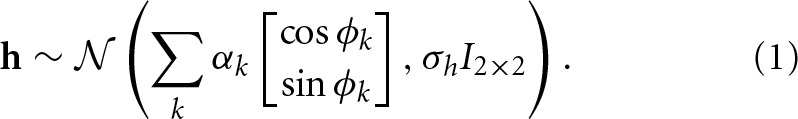


The regression coefficients are directly interpretable. They give the magnitude of the displacement from the reference point due to each factor in degrees. Again, the dart's concave vertex and circle's center are used as reference point.

Once more we use hierarchical priors to pool the estimates of the regression coefficients across participants in order to obtain the population estimates. The detailed formulation of the model is given by S1.1 in Supplementary Table 2.

#### 3.3.2. Bugs

Figure 8 shows how the various factors influence the displacement in recall. There is no indication of representational gravity or momentum. The hand inertia and agent orientation are equally strong and confounded. As a consequence, the data in C and D are shifted diagonally. In C, on half of the trials the agent is pointing in the same direction as the hand inertia. On the other half of trials the agent is oriented in the negative direction of the vertical axis. To disentangle the influence of the two factors we look at the estimates from the regression model. The estimates of the regression coefficients are given in Table 2 (R1.1). Of most interest to us, the displacement due to orientation is positive (the recalled positions are shifted in the direction of the eyes), albeit the 95% interval includes zero.

**Table 2:** **Displacement in location recall**.

**Agent**	**Gravity**	**Momentum**	**Hand inertia**	**Orientation**	**Slope**	**Analysis**
Bug	0.01 [−0.2, 0.23]	−0.02 [−0.15, 0.1]	0.26 [−0.03, 0.54]	0.14 [0, 0.28]	–	R1.1
Dart	^*^	0.04 [−0.05, 0.13]	0.38 [0.22, 0.54]	0.12 [0.03, 0.21]	–	R1.2
Dart	^*^	0.03 [−0.05, 0.09]	0.48 [0.32, 0.64]	0.14 [0.06, 0.22]	1.07 [0.84, 1.311]	R1.4
Circles	−0.01 [−0.18, 0.17]	0 [−0.09, 0.1]	0.38 [0.14, 0.63]	0.01 [−0.11, 0.15]	1.16 [0.41, 1.9]	R1.5

*The values in brackets give the 95% interval*.

* *—The gravity estimates were positive (i.e., pointing upwards) which is implausible. Since the positive estimates were not reliably different from zero we removed gravity as a predictor from the analysis. This was done in accord with the recommendations for regression analysis in Gelman and Hill (2007)*.

#### 3.3.3. Darts

**Figure 9:**
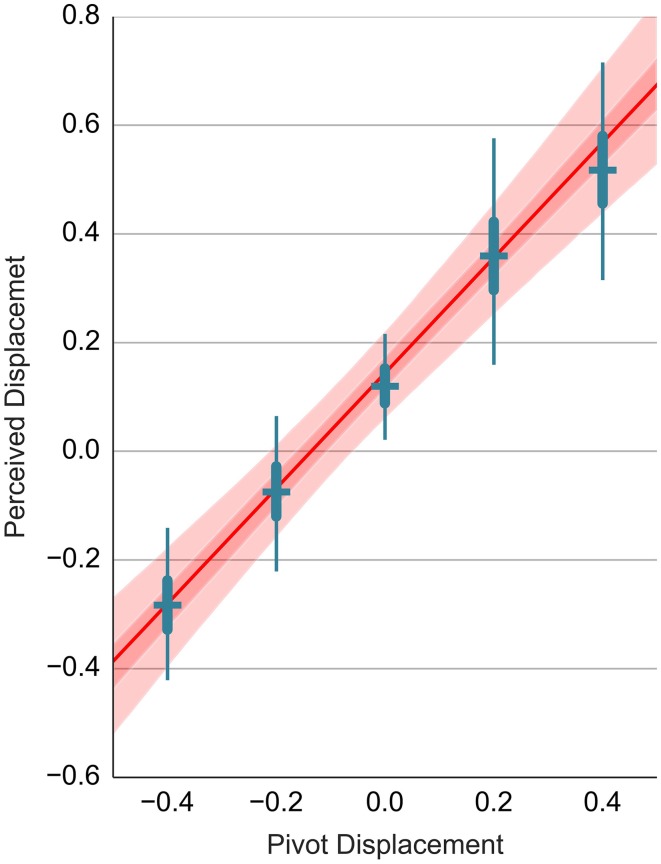
**Relationship between the perceived and the pivot displacement in the Location Recall task**. This figure uses the same layout as Figure 6. Refer to the caption of Figure 6 for details.

As with the analysis of the Distance Bisection task with the darts, we first group the data according to where the pivot was placed (Figure 2) and estimate a separate model for each condition (R1.3). Then, we analyze all data together by adding the pivot shift as a predictor of the orientation displacement (R1.4). In particular, α_4_ = μ_α,4_ + β_α,4_*d*, where *d* is the displacement and β_α,4_ is the slope coefficient (see S1.2 for details). However, with this model we obtained a regression line with a slope of 0.79, 95% PI [0.57, 1.02] that inadequately fitted the data from trials with non-zero pivot shift. This was caused by the data from trials with pivot at zero (i.e., from the first block), which, due to the high precision and slightly lower offset, pulled the regression line downwards. We omitted these trials from line fitting and fitted a separate model (S1.1) to the data with zero pivot displacement (R1.2). The results are shown in Figure 9 and the estimates are given in Table 2. As expected, the slope is around 1 and we observe positive constant displacement due to the dart's orientation. Similar magnitude of the perceived displacement is obtained for the trials with pivot at zero (R1.2).

#### 3.3.4. Circles

For the sake of completeness, the results of fitting the regression model (R1.4) with the circles are shown in the last row of Table 2. As expected, the perceived displacement varies with the pivot position. Due to the small sample size the estimates are very noisy. Still, we observe displacement due to gravity, motion and hand inertia of a similar magnitude to that observed with the darts and the bugs.

### 3.4. LMA task

#### 3.4.1. Statistical modeling

Gao et al. (2010) computed the proportion of the time spent in the wolfpack quadrants on each trial. They then computed the average proportion for each participant and showed, with a *t*-test, that the participant averages are significantly lower than 0.5, where 0.5 is the expected proportion if the participants show no preference.

Such analysis is problematic because it discards the within-participant variability. Taking the mean across the proportions from individual trials is only valid if the proportions from trials are normally distributed with the same standard deviation for each participant. We use hierarchical modeling to account for within-participant variability as we did in our previous analyses. As in Gao et al. (2010), we compute the proportion of time spent in the wolfpack quadrants on each trial. This is a quantity in the range between 0 and 1. In principle, we could model these fractions with a (truncated) Gaussian model. However, for several participants the distribution of the measured fractions is virtually flat, which makes it impossible to fit a Gaussian distribution. Instead, we use beta distribution parameterized by mean proportion and sample size to model within participant variability. In addition, we use beta distribution to model the variability across participants (S2.1). Alternatively, we explored a hierarchical Gaussian model that was fit to the data transformed by logit function. Both the beta and the logit-normal model fit the data well. Furthermore, the two models give practically identical estimates for the variables of interest. We report the results from the beta model since its parameters are easier to interpret.

#### 3.4.2. Bugs

**Figure 10:**
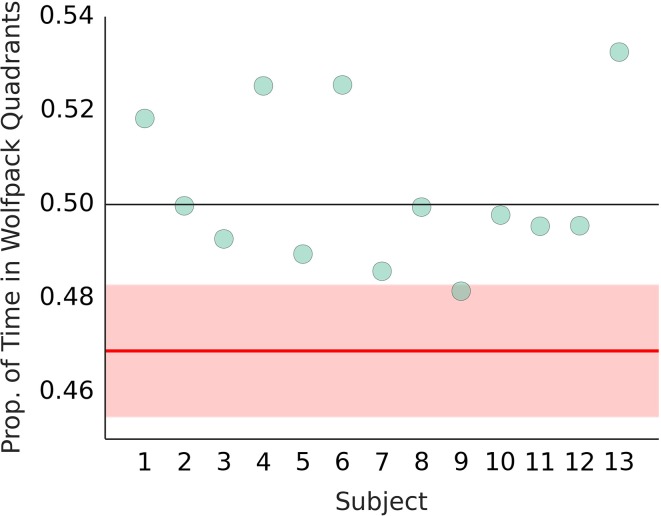
**Wolfpack avoidance with bug stimuli**. Dots show the average proportion of time spent in the wolfpack quadrants by participants in our sample. The red line shows the mean estimate reported in Gao et al. (2010) and the red surface shows the 95% CI. Black line at 0.5 shows the expected chance performance.

We did not observe the avoidance of the wolfpack areas reported in Experiment 3b in Gao et al. (2010). Figure 10 shows the 95% confidence interval based on the analyses reported in Gao et al. (2010) along with the averages for each of our participants. All but one participant were located above the upper boundary of the confidence interval. Using the hierarchical beta model, we obtained a mean estimate of 0.504, 95% CI [0.482, 0.525] for the proportion of the time spent in the wolfpack quadrants (R2.1). The mean estimate of 0.469 reported by Gao et al. (2010) is inconsistent with our data.

#### 3.4.3. Darts

**Figure 11:**
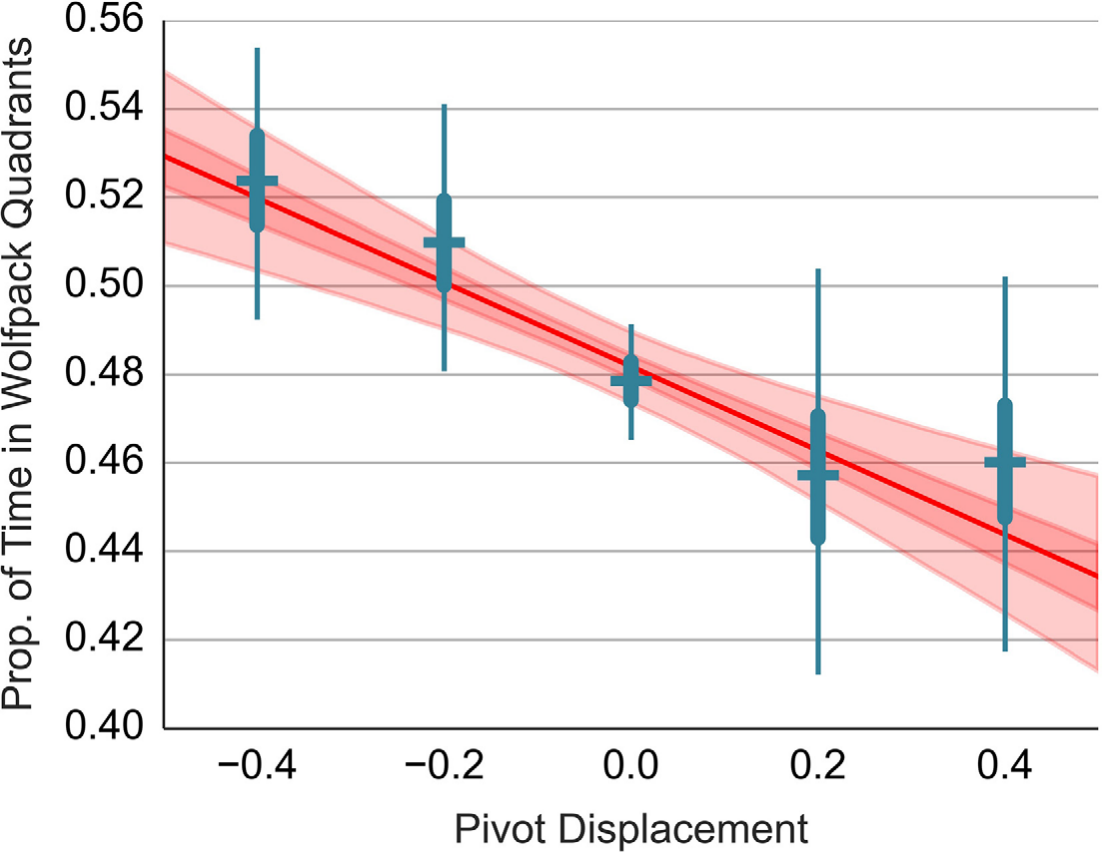
**Relationship between the wolfpack avoidance and the pivot displacement**. This figure uses the same layout as Figure 6. Refer to the caption of Figure 6 for details.

As with previous models, we now include the pivot displacement as a linear predictor into the beta model (S2.2). Again we evaluate and plot the slope of the regression line together with the estimates for sets of trials grouped by the pivot displacement. The results are shown in Figure 11. In the trials with pivot at zero, the mean proportion estimate is 0.478, 95% PI [0.466, 0.49] (R2.2). The estimate of the pivot at zero from the regression model is 0.482, 95% PI [0.474, 0.489] (R2.4). If we shift the pivot by one degree away from the nose, the mean proportion of the time spent in the wolfpack quadrants decreases by 0.093, 95% PI [0.053, 0.132] percent. That is, if we shift the pivot toward the nose, the wolfpack effect in the LMA task becomes smaller. We can compute an estimate of the pivot shift required in order for the wolfpack effect to disappear. The estimate is given by (0.5−μ_μ_)/μ_β_ = 0.2, 95% PI [0.1, 0.34], where μ_μ_ is the intercept and μ_β_ is the slope obtained by the regression model (S2.2). This estimate is consistent with the perceived displacement observed in other tasks. If we shift the pivot to where the participants perceive the center of the agent's body in the Location Recall and the Distance Bisection task, the wolfpack effect disappears!

Finally, we observe once more a sigmoidal pattern in the individual estimates. This shows that the linear model is not a good model for the data at hand.

#### 3.4.4. Circles

We fitted the regression model to the data from the LMA task obtained with circles (R2.5). The slope was estimated at −0.018, 95% PI [−0.142, 0.104]. The pivot displacement does not reliably influence the preference for the wolfpack quadrants. However, the slope estimate is very imprecise and does not allow us to reliably exclude that such influence exists.

### 3.5. Modeling the mouse movement from LMA task

In addition to the data discussed so far, we also obtained measurements of the mouse position on the screen for each participant and for each trial of LMA task sampled at 75 Hz. We use a simple force field model to account for the participant's choice of mouse position in trials where the pivot was set to zero. We then use this model to infer the perceived displacement.

An agent *a* located at position **g** = [*g_x_, g_y_*] emanates a repulsive force at location **h** = [*h_x_, h_y_*] given by a vector $F_{a}(h)=\frac{1}{r^{2}}[\cos\phi ,\sin\phi ]$ where ϕ is the angle of the vector pointing from **g** toward **h** and *r* is the distance between the two points. This definition is identical (except for the vector sign) to the definition of gravitational force in physics. In addition to avoiding contact with the distractors many participants avoid the circular boundary. We therefore added a force that emanates from the boundary. It is given by $F_{\text{boundary}}(h)=\frac{1}{r^{2}-12}[\cos\phi ,\;\sin\phi ]$, where ϕ and *r* are defined as above with **g** = [0, 0]. We propose that in order to avoid contact, participants minimize the net force. Net force is the vector sum of the forces created by the agents and the boundary *F*_net_(**h**) = *cF*_boundary_(**h**) + ∑_a_ *F_a_*(**h**). *c* is a parameter that determines the relative contribution of the boundary to the net force.

**Figure 12:**
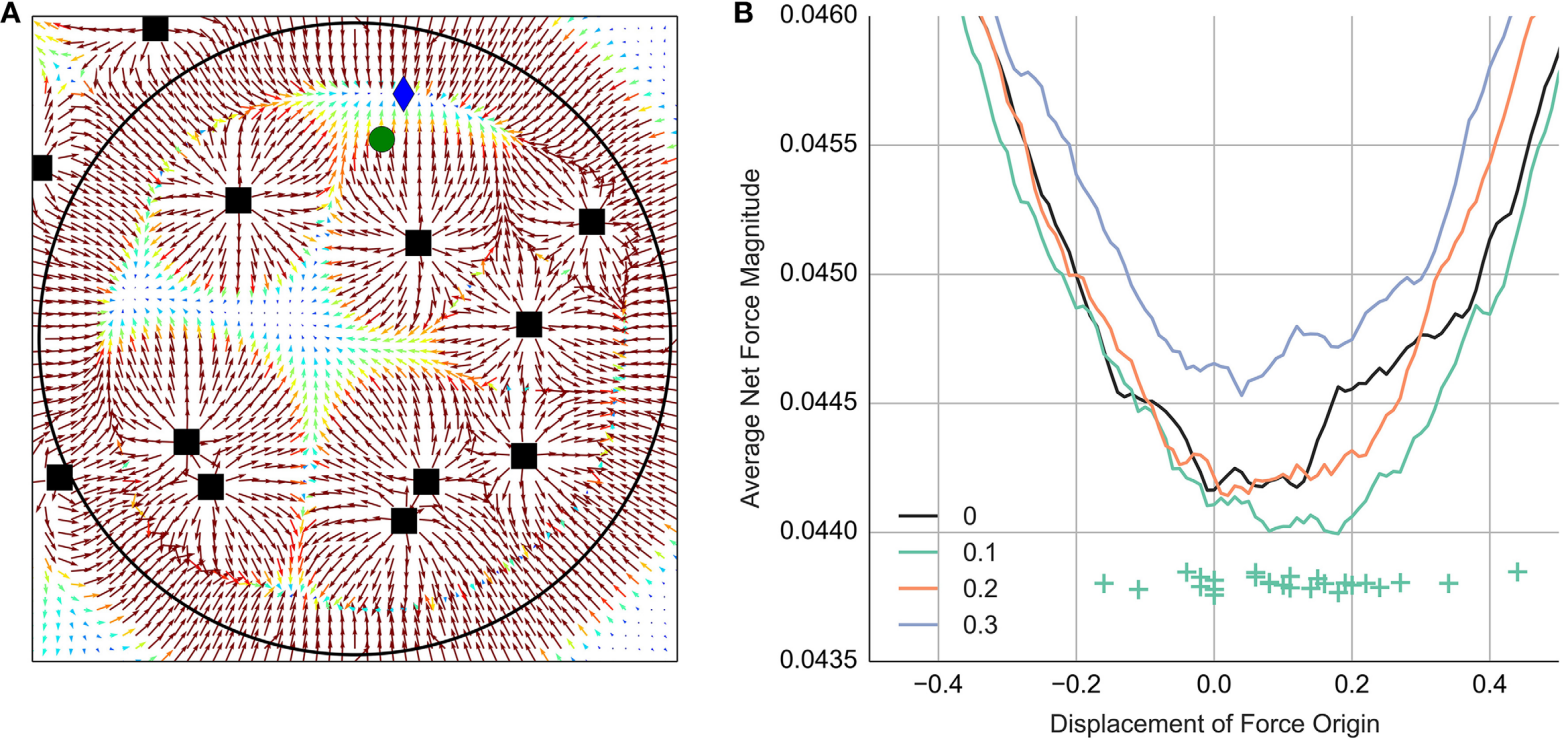
**Modeling the mouse movement**. **(A)** Shows a visualization of the force field as described in the text. Black squares show the agents' position. Position of the green circle was controlled by the participant. The arrows show the net force at each location *F*_net_(**h**). The magnitude of the net force vectors is highlighted in color, with hot colors corresponding to strong force. For the purpose of visualization the force magnitude is capped at 0.1. The blue diamond shows the position of the nearest net force minimum reached by following the arrows from the green circle. **(B)** Shows how the average net force magnitude at the position of the green circle varies for different values of displacement. The net force was computed either at 0, 0.1, 0.2 or 0.3 s after the event onset. The crosses show the position of the minimum for each participant when computed 0.1 s after the event onset.

Figure 12A shows an example of the force field. Net force minimization can be done by moving the green circle in the direction in which the net force is pointing. Participants stop moving at locations where the net force is zero. The blue diamond-shaped marker in Figure 12A shows the point with zero net force reached by following the arrows from the green circle's location. Note, the shape of the force field changes constantly as the agents continue to move around the screen. These changes can be seen in a movie at http://vimeo.com/89325231.

We analyze the net force at the position of the green circle controlled by the participant. We compute average net force across frames, trials and participants. If participants minimize the net force and if they perceive the agent's body as displaced in the direction of its orientation, then the net force should be smallest when the force origin is set to the perceived center of the agent's body. To simulate the displacement of the agent's body we shift the agent's force origin in the direction of its nose. We try out multiple shift values to see what degree of displacement best describes participant's judgments.

The details of the analysis are as follows. We do not wish to measure the net force at the intervals when participants make a quick movement to a new location. We therefore isolate events during which participants are satisfied with their current position. To isolate these events, we compute the velocity at each frame. We then smooth this times series with a Gaussian filter with standard deviation of one frame. Consecutive frames with a velocity below 15 degrees per second and a duration of at least 300 ms qualify as an event. We measure the net force at the position of the green circle (**h**) at the start of each event and 100, 200 and 300 ms after the event onset. To account for the lag between the perception and the execution of the mouse movement we compute the net force based on the agents' location (**g**) 200 ms before the mouse movement, i.e., −200, −100, 0, and 100 ms with respect to the event onset. We discard the first event at the start of each trial, since this location was generated by the computer program. We compute the magnitude of the net force vector at the mouse position |*F*_net_(**h**)|. We compute the median magnitude across all trials and events for each participant. We set *c* for each participant individually to a value with minimum median magnitude (found by grid search). The differences in *c* reflect different strategies used by different participants. While some participants prefer to stay in the middle of the screen (large *c*), other pay frequent visits to the borders of the movement area (small *c*).

Figure 12B shows the median net force magnitude averaged across participants for different displacement values and different time offset from the event start. Measurements at 100 ms after the event onset show the smallest net force. In this particular case the basin of the minimum net force indicates a positive displacement in the range between 0.05 and 0.2 degrees. Unfortunately, we lack a probability model that would tell us how big the observed differences in the net force are compared to the noise in our observations. We can make a tentative estimate by analyzing the median displacement of each participant. The crosses show the net force minimum for each participant measured 100 ms after the event onset. Then using the Student's *t* distribution to model error, we obtain a mean displacement of 0.13, 95% CI [0.06, 0.19] degrees.

We presented results for parameter values that minimize the net force and thus provide the most reasonable case if we assume that participants aim to minimize the net force. The obtained estimates are all reasonable, which in turn provides some convergent evidence that participants do indeed minimize the net force at least to some degree. For readers who remain skeptical we add that the basin between 0 and 0.2 degrees displacement is quite robust to different decisions. As one can see in Figure 12B, the basin does not change much with different choices of the relative time point. Further analyses (not reported here) showed that the basin between 0 and 0.2 is also obtained if we take samples at constant intervals (instead of computing events) or if we work with mean instead of median statistics.

### 3.6. Comparison between tasks

**Figure 13:**
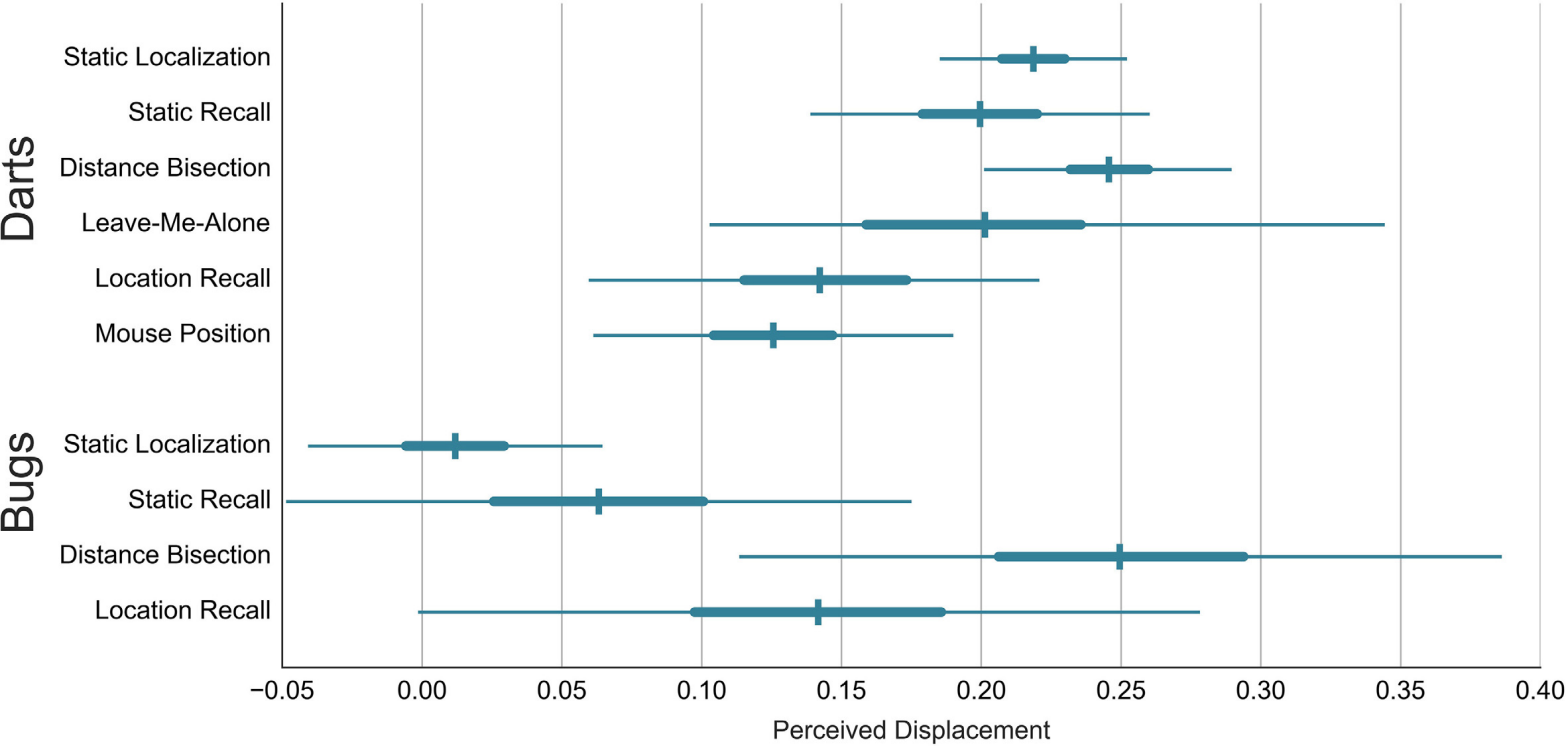
**Comparison of the perceived displacement across tasks and stimuli**. Error bars show the mean estimate and the 50 and 95% interval.

So far we have considered the results from each task individually. We now compare the perceived displacement between tasks. A summary of the results reported so far is provided in Figure 13. Two comparisons are notable. First, the agent's center selected by the participants for static bugs is inconsistent with the perceived displacement in the Distance Bisection task. Second, the perceived displacement in the Distance Bisection is stronger than in the Location Recall task (especially with darts).

Another way to compare the tasks is to compare the performance for individual participants and to see whether participant's performance in one task is similar to the performance in other tasks. We do this by giving the participant-level parameters from different tasks a common multivariate normal hierarchical prior. We use here only data from the Location Recall, the LMA and the Distance Bisection task since for the other measures shown in Figure 13 we lack a model of within-participant variability. Furthermore, we analyze only the data from participants who saw darts and from trials where the pivot was at zero.

The data from the location displacement task are modeled as in S1.1. The data from the Distance Bisection task are modeled as in S3.1. For the LMA task we use a logit-normal model instead of the beta model S2.1. In particular, logit(*w_t, i_*) ~ 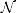 (γ_*i*_, σ), where *w_t, i_* is the proportion of time spent in the wolfpack quadrants by participant *i* on trial *t*. Then we define the hierarchical prior for α_3, *i*_, μ_*x, i*_ and γ_*i*_ as 
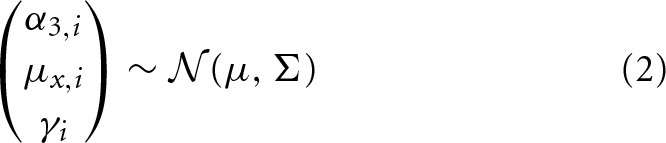
 where Σ is the covariance matrix given by

(3) $$\left(\begin{array}{ccc} \sigma_{l}^{2} & \sigma_{l}r_{lw}\sigma_{w} & \sigma_{l}r_{lb}\sigma_{b}\\ \sigma_{l}r_{lw}\sigma_{w} & \sigma_{w}^{2} & \sigma_{b}r_{wb}\sigma_{w}\\ \sigma_{l}r_{lb}\sigma_{b} & \sigma_{b}r_{wb}\sigma_{w} & \sigma_{b}^{2} \end{array}\right)$$

We are interested in the estimates of the correlation coefficients *r_lb_, r_wb_*, and *r_lw_*, where *l, w, b* indicate location recall, LMA task and Distance Bisection task, respectively. While the model fits the data well with estimates very similar to the estimates obtained from the separate models, the correlation coefficients could not be estimated reliably. Precisely, *r_lb_* = 0, 95% PI [−0.84, 0.85], *r_wb_* = 0.03, 95% PI [−0.82, 0.87] and *r_lw_* = −0.01, 95% PI [−0.89, 0.87]. The 95% interval estimates are essentially identical to what one would expect under the uniform prior specification (i.e., 95% PI [−0.9, 0.9]). The data thus provide little information about the correlation between the participants performance in the three tasks. By performing a fake data simulation (see chapter 16.7 in Gelman and Hill, 2007) we determined that we would require at least 200 participants to cut the standard error of the correlation parameters by half.

Before closing this section, we can make one additional comparison. Recall that when judging the static stimuli one participant put the center at the nose of the dart (dark purple cross in Figures 4D,E). If this participant did also perceive such a huge displacement (median 0.76) in other tasks, this should be readily detectable even with our imprecise participant-level estimates. However, no such displacement does occur in the other tasks. The displacement for this participant is 0.04, 95% PI [−16.97, 0.45] in the LMA task, 0.04, 95% PI [−0.48, 0.75] in the Location Recall and 0.23, 95% PI [−0.06, 0.52] in the Distance Bisection task. Thus, even though the displacement in darts is similar between static and dynamic stimuli, the case of the dark purple participant suggests that this correspondence is coincidental and that with the darts, as with the bugs, the explicit and the implicit judgments are dissociated.

## 4. Discussion

The results from the Location Recall task and the Distance Bisection task are inconsistent with the position where Gao et al. (2010) put the pivot. With darts and with bugs, the perceived center is shifted in the direction in which the agent is facing. This supports our first claim.

In support of our second claim we have shown that with the darts, the performance in the LMA task changes if we shift the pivot. The direction and the magnitude of this shift is consistent with our second claim. Notably, the avoidance of wolfpack areas disappears if we shift the pivot to the center of the agent's body. Several observations complicate such interpretation of the results and we discuss these complications next.

### 4.1. Trial order

We observed two instances where the pivot displacement resulted in a sigmoidal shape of the outcome variable. This was the case with the avoidance of the wolfpack areas (Figure 11) and with the vertical displacement in the Distance Bisection task (Figure 6B). We offer the following explanation why the relationship is not linear.

We think the non-linear shape is due to the choice of the experimental design. If we had presented the trials with different pivot displacement in separate blocks and not randomly shuffled, we would have obtained a linear relationship.

Consider the LMA task. The participants were not told that the pivot displacement would vary throughout the block. If one has to decide where to move on the screen, one needs not only to estimate the advantage (in terms of avoiding being caught) of staying in the current area, but also the advantage of moving to some alternative area and staying there. While the former information should be readily available from looking at the screen, the latter requires inference of what will happen once the chasee moves to a different area. How dangerous are the agents there? How will they react to chasee's presence? Will they advance toward the chasee and by how much? It is plausible that participants estimate some degree of threat posed by an average agent. It is also plausible that this depends on where the agent is located with respect to pivot. Wolfpack agents behind the pivot will be less threatening than the agents with the body leaning out in front of the pivot. What happens if the threat level varies randomly across trials? Participants will estimate a threat level that is an across-trial average. The observed decisions will thus be influenced by two factors. First, by a direct perception of the danger in the current area, which is influenced by the pivot displacement only in the current trial. Second, by a predicted estimate of the threat level in other areas. The latter will be based on the evidence from the pivot displacement in other trials and will thus mix evidence from different conditions with different pivot positions. The latter factor will make the decisions more similar. Thus, rather than displacing pivot by {0.4, 0.2} and {−0.4, −0.2} our data should be interpreted as displacing pivot by {0.3 + some, 0.3−some} and {−0.3−some, −0.3 + some}. If we would re-plot the estimates at these values we obtain the expected linear relationship.

We can give a similar explanation for the results in the Distance Bisection task. The instruction was asymmetric with respect to head-on and perpendicular displacement. While the participants adapted their position based on the position of the head-on agent in real-time, they corrected the position with respect to the perpendicular agent only occasionally - once the discrepancy was too big. The less tight temporal coupling gives the participant an opportunity to utilize prediction mechanisms that mix information across trials which would lead to the sigmoidal pattern of results. Note however that the sigmoidal shape is not present in Figures 6A, 9. This is to be expected since the participants here can't rely on any inferential processes that would accumulate evidence across trials.

Do such trial order effects challenge our interpretation of the results in Figure 11 with respect to our second claim? The trial order effects add an additional source of noise so that the slope estimate is less precise. As stated above, instead of two conditions with absolute pivot shift of {0.2, 0.4} we have one mixed condition with an average 0.3 shift. With data from separate blocks with pivot at −0.3, 0, 0.3 we can still make a reasonable estimate. The situation is much more problematic in the case of the experiments with the bugs. Here we mixed up conditions with negative and positive displacement. If the participants indeed average the evidence across trials, then the effects of the positive and the negative pivot shift cancel out, such that we obtain pivot near zero on average. We think this is the reason why we did not obtain a slope reliably different from zero in the LMA task with displaced circles.

A reader may feel irritated, that we waste his or her time with accounts of our problems with experiment design. We should have run another sample of participants with conditions blocked together and then report the results of these solid experiments. This discussion of trial order effects would then be superfluous. However, we think this discussion is actually instructive because it pertains to other published studies. In the supplement we discuss how trial order effects may complicate the interpretation of the experiments in Gao and Scholl (2011).

### 4.2. Unsuccessful replication

We did not find wolfpack avoidance in the LMA task with the bugs. After completing the experiment sessions where we tested participants with the bugs, we looked at the participants' behavior in more detail. The analyses suggested that participants who travel longer distances (with the green circle) are better at avoiding collisions and also, at least to some degree, show the wolfpack effect. We wanted to ensure that our failure to replicate is not because our participants were slacking at the task. Hence in the experiments with the darts we introduced a beep tone which was sounded at contact. This definitely made the task more engaging with several participants cursing throughout the first two blocks. We think that the missing beep tone and not the stimulus was the reason for our failure to find a wolfpack effect with the bugs. Gao et al. (2010) did not use any sound feedback, however they excluded participants who did not move around the display (less than 10%).

This failure did not allow us to explore the influence of the pivot displacement on the wolfpack avoidance with circles which falls under our second claim. However, we have provided evidence for the second claim in our experiments with the darts. We do not see any reason why with the darts the wolfpack avoidance should be due to the perceived displacement while with the bugs there should be some totally different factor at play. The experiments with the bugs/circles are crucial with respect to our first claim because, as discussed in the introduction, there are good reasons why these stimuli should differ in the terms of the perceived displacement. This claim was supported.

### 4.3. Displacement across tasks

While the results consistently show that the pivot point used by Gao et al. (2010) is not the perceived center of the agent's body, there are also considerable differences between how much the center is displaced in the different tasks in our study. Which task provides the best estimate of the displacement?

We favor the head-on displacement from the Distance Bisection task. As discussed in the introduction, the judgments in the Location Recall task are susceptible to correction and this was the primary reason why we included the Distance Bisection task. We expected that participants would correct their judgments in the Location Recall task toward the position of the explicit judgment, i.e., the position indicated in the experiments with the static stimuli. The results from the Location Recall task with the bugs are consistent with this idea. The magnitude of displacement is a compromise between the displacement in the task with the static stimuli and the displacement in the Distance Bisection task. With darts however, the explicit judgment is similar to the head-on displacement, but the displacement in the Location Recall task is nonetheless pulled toward the concave vertex.

One possible explanation is that participants were not oblivious to where we put the pivot. Eventually, when a dart rotated, participants would notice the pivot's position, especially if the green circle made a rapid movement in the vicinity of the agent. If this is the case, we should expect a correction toward zero in the trials where the pivot displacement is {0.2, 0.4} and no correction in the trials where the pivot displacement is similar to the perceived displacement. That is {0.2, 0.4} trials should show smaller constant perceived displacement than {−0.4, −0.2} trials (while the slope should remain around one in both cases). But looking at Figure 9 we see no such pattern. All conditions fit the regression line (slope 1.07) well.

The only explanation we can think of is that the estimates of the magnitude of the hand inertia and the magnitude of the orientation displacement interact. This would violate the assumption of linear additivity used in our analysis. The resulting displacement may not be the linear sum of the two factors. For instance, the participants may correct their judgment only if the displacement between the perceived and the actual position exceeds some threshold. In such a case, they would make larger correction for wolfpack stimuli where the orientation and hand inertia point in the same direction. Consequently, a correction of hand inertia may influence the estimate of the orientation displacement and in particular pull it toward zero. This issue could be readily resolved by experimentally decoupling the agent's orientation and the position of the chasee or by omitting the green circle altogether.

### 4.4. Displacement across stimuli

We observed different displacement with the static darts and the static bugs. This difference points to a common factor, namely the position of the center of mass. However, in darts, the displacement was located further toward the nose than the center of mass. The explicit judgment thus probably reflects the intuitive physics (McCloskey, 1983) rather than a veridical computation.

If we make a comparison of the displacement in dynamic stimuli we see that bugs and darts are similar. This brings us to an intriguing thesis. Maybe the displacement is not influenced by the visual properties of the stimulus such as a pointed shape or some surface features. The displacement may be driven by the knowledge that the stimulus is an agent. In such a case we should expect a constant displacement for all agents irrespective of their shape and surface properties (but possibly scaled by their overall size). This could be tested by manipulating participants' beliefs about the orientation of the anteroposterior axis of the agent with respect to the visual features. For instance, what happens if we tell participants that the bugs are facing in the direction opposite to the orientation of the “eyes”? In which direction will the displacement go?

Our experiments suggest that the link from perceived displacement to explicit judgment is not very strong (recall the comparison in Figure 4 between D, E and F, G). Plausibly the link would not be very strong the other way round from explicit knowledge to the perceived displacement (though we did not want to risk this possibility and for this reason we presented the trials with the static stimuli at the end of the session). Furthermore, the literature on memory displacement suggests that displacement does not reflect explicit knowledge (Freyd and Jones, 1994). As a consequence, instead of just telling participants which direction the agent faces, the proper manipulation would be to let participants interact with the agents in some dynamic competitive task. How would a different orientation of the anteroposterior axis with respect to the visual features in the training influence the perceived displacement in the subsequent tasks?

### 4.5. Displacement across factors

In the Location Recall task we observed orientation displacement and hand inertia but no representational gravity, nor representational momentum. Here we discuss these results in connection with the literature on memory displacement.

The displacement in gravity and momentum consistently showed a magnitude around zero for both stimuli (see Table 2). For comparison, Hubbard and Bharucha (1988) reported displacement of 0.03 degrees for stimuli moving in the bottom-to-top direction and 0.08 for horizontally moving stimuli^5^. Our results do not exclude the possibility of such small displacement.

Hand inertia as operationalized in our experiment does not qualify as a memory displacement factor. It represents the motivational and motor factors that influence the proportion of the distance that the participants are willing to travel with the mouse cursor toward the target location. Furthermore, Müsseler et al., (1999) have shown that the recalled location is displaced toward the gaze location. Throughout the LMA task, participants would track the green circle or look at the potential escape locations in its vicinity. The pull toward the gaze location would then add to the magnitude of the hand inertia. It is probably due to the accumulated influence of all these factors that we observed such a large magnitude of hand inertia in our experiments. Looking at Table 2 we note that hand inertia is larger in the second block and is larger with darts than with bugs. Participants probably devoted more attention to the Location Recall task with bugs than with darts (because of the tone feedback in the latter case). They were also probably more concentrated in the first block than in the second block. This makes sense if hand inertia increases when participant's concentration decreases.

What we call orientation displacement with darts has been previously studied as influence of shape pointedness on representational momentum. Freyd and Pantzer (1995) showed that a pointed shape of an arrow enhances representational momentum if the arrow points in the direction of the motion and has an attenuating effect if the arrow points in the opposite direction. Nagai and Yagi (2001) presented similar results for shapes of airplanes. Vinson and Reed (2002) investigated the interaction between the pointedness and the conceptual knowledge about the object. They suggested that the identity of the object is more important than its shape, especially if the object is a prototype of its category. This is consistent with our finding of displacement with the bugs which provide no shape information. Recall however that we did not find a displacement with static bugs. It is possible that the static bug failed to engage the relevant conceptual category.

Freyd and Miller (1992) showed that the orientation of an abstract creature influences the displacement as compared to the same stimulus but with its parts scrambled. On the other hand, Halpern and Kelly (1993) compared the displacement in drawings of animate (e.g., fox) and inanimate (e.g., truck) objects and did not find any differences. Their results suggest that agency rather than animacy is the critical cue. In support of this Hubbard and Ruppel (2002) showed that displacement was smaller if the target was pushed into motion by another self-propelled object. Our results support this interpretation. Participants interpreted darts and bugs as agents but not necessarily as animate. For instance, one participant spontaneously referred to the darts as airplanes.

There are also some notable differences that set the orientation displacement apart from the existing literature on memory displacement. The magnitude of orientation displacement is larger than the magnitude usually reported in the other studies. The location recall was immediately preceded by the LMA task. On the one hand, with bugs this may have depleted the resources that would be used for correcting the displacement in Location Recall. On the other hand, as we explained in the introduction, the LMA task would provide a dynamic context in which the orientation cue becomes highly relevant for the prediction of motion. As hypothesized by Hubbard (2005), in such a case we would expect large displacement. If this account by Hubbard (2005) is correct, it suggests that providing dynamic interactive context may be a fruitful avenue for memory displacement research. Furthermore, as our results from the Distance Bisection task suggest, the phenomenon goes beyond the displacement in memory and should be referred to as displacement.

### 4.6. Is there an orientation cue to goal-directed motion?

Gao et al. (2010) used the results from their experiments to argue for two claims. First, they claimed that the wolfpack effect is a novel cue to perceiving animacy. Second, they claimed that this cue was irrelevant to the participant's task and that as such, the effectiveness of this cue represents automatic and reflexive perceptual mechanisms. Our results indicate that the wolfpack effect is not a novel cue but consists of effects of subtle chasee-directed motion aggregated across a number of agents. Furthermore, this motion was highly relevant to all tasks in Gao et al. (2010). In the introduction, we already used this fact to devise alternative explanations of the results of their experiment 3 and 4. The performance in these experiments could be accounted for by domain-general processes such as distance estimation and distance maximization or net force minimization.

In experiment 2 (and 1) in Gao et al. (2010) the motion of distractors was not strictly relevant to the task. However, if the participants were searching for a heat-seeking motion toward the chasee, the subtle motion of distractors might have drawn selective attention and as a consequence compromised their performance. If the participant's attention was selectively focused to a chasing motion, the darts in the wolfpack condition would distract, while the darts in the perpendicular condition would be neutral. In face of these alternative explanations we do not think that the wolfpack effect is anything more than a subtle motion cue. Neither do we think that the results in Gao et al. (2010) support the notion of perceptual animacy as automatic, reflexive or irresistible (Scholl and Gao, 2013).

Finally, we note that the participants in Gao et al. (2010) described the agents' behavior as motion toward the chasee rather than a rotation. In the light of these observations we do not think that it is necessary to postulate orientation as an additional and separate cue that supports perception of goal-directed motion.

### 4.7. How to study performance in interactive tasks

We close by discussing an alternative interpretation of how perceived displacement arises and the consequences of this interpretation for the future research. The term perceived displacement may be slightly misleading. It suggests that the displacement arises during perception and as such is predominantly influenced by the properties of the stimulus such as shape or direction of motion. However, the perceived displacement probably arises in large part during interaction of the participant with the task. In such a case people do not represent, not even implicitly, the position of the center of the agent's body. Rather they develop a strategy how to efficiently solve the task. The task context is negligible in the traditional experiments on memory displacement. However, the context becomes relevant when the task is dynamic and interactive. The results from the tasks with static stimuli show that people do have some idea about the displaced center. We also saw that the explicit measures are dissociated from the implicit measures. Looking at implicit tasks we saw that the magnitude of the displacement varies considerably across the tasks, while the displacement is very similar across stimuli. This would be expected if the task context and the task related strategy is the main factor influencing the displacement.

If we acknowledge the contribution of the task context, it does not make much sense to ask what is the true displacement as we did in Section Displacement across tasks. In the context of this study, we prefer to view perceived displacement as a psychological construct which primary purpose was to highlight the problems with the claims in the study by Gao et al. (2010). Future research should focus on the precise mechanisms that give rise to the displacement in the various tasks.

Acknowledging that task demands are important has also implications for future research practice. First, we need to look at the human interaction with the task at much finer time-scale at which the precise mechanisms become distinguishable. Second, we need to consider the behavior of a participant as a factor that potentially influences (later) performance. At the same time we need to keep the dynamic interactive context intact. That means that we can't manipulate participant's actions, at least not directly. The solution is to rely more heavily on observational studies. Both issues can be helped by a model-based analysis. To study performance at fine time-scale we need complex formal models that link the measured phenomena to the abstract psychological constructs. Formal models can further help digest observational data e.g., by correcting for confounds, that cannot be eliminated by experimental manipulation. Our analysis of the mouse movement data showed how model-based approach may work. The displacement estimation does not require any manipulation or a particular experimental design. The displays may even include different classes of distractors and the displacement created by each of them can be separated in the analysis. Furthermore, the model can be fruitfully extended by other factors that influence the performance such as learning or participant's choice of strategy. In our model, we accounted for different choice of strategy by adding repulsive force that emanates from the circular boundary.

In sum, we think the future study of participant's performance in dynamic interactive tasks should focus on observational studies supplemented by model-based analysis.

## Author contributions

Matúš Šimkovic conceived research, conducted experiments, analyzed data, and wrote manuscript. Birgit Träuble conceived research and wrote manuscript.

## Funding

The research was supported by Grant TR898/6-1 from the Deutsche Forschungsgemeinschaft to Birgit Träuble.

### Conflict of interest statement

The authors declare that the research was conducted in the absence of any commercial or financial relationships that could be construed as a potential conflict of interest.

## Footnotes

^1^Demonstration movie from the authors is available at http://tinyurl.com/kcdckbs

^2^Demonstration movie from the authors is available at http://tinyurl.com/lxunkrr

^3^Demonstration movie from the authors is available at http://tinyurl.com/n53rs7n

^4^The analyses were originally performed with STAN 2.0.1. According to STAN development team there was a bug in 2.0.1, due to which the uncertainty of parameter estimates may be underestimated. We repeated the analyses with STAN 2.2.0, these are included in the file Revision1.ipynb in the repository and are reported here.

^5^Representational Momentum increases with velocity of the stimulus. Hubbard and Bharucha (1988) used 5.8 degrees per second. We used 7.8. These values are reasonably similar to allow comparison.
