# Supplementary material for: Perceived displacement explains wolfpack effect
Source: Front Psychol. 2014 Dec 16;5:1423. doi: 10.3389/fpsyg.2014.01423 (PMC4270252; doi:10.3389/fpsyg.2014.01423)
Supplement: Supplementary file 1 [file DataSheet1.PDF]

Frontiers in Psychology

Research Article
20 October 2014

Supplementary Material: Perceived Displacement explains Wolfpack Effect

Matúš Šimkovic [1,*] and Birgit Träuble [2]

[1,2] *Universität Heidelberg, Germany*

Correspondence*:
Matúš Šimkovic
Universität Heidelberg, Hauptstr. 47-51, D-69117, Heidelberg,
matus.simkovic@psychologie.uni-heidelberg.de

1 AN EXAMPLE WHERE TRIAL ORDER EFFECTS POTENTIALLY CONFOUND THE INTERPRETATION

As an example consider the experiments in **Gao and Scholl** (2011). Here the authors presented a chaser that moved in heat-seeking manner only some of the time and moved randomly for the rest of the time. The exact proportion was varied at $0, 0.2, 0.4, 0.6, 0.8, 1$. Subjects tried to detect and escape the chaser among a field of randomly moving distractors. They found that the success rate was 0.55 [1] with the fully random chaser and 0.45 with the fully heat-seeking chaser. However, they observed a success rate of 0.2 for the other four conditions with intermediate values. The authors interpreted this dramatic decrease in the performance as a hallmark of human visual processing. However, since the trials were presented equiproprotionaly and randomly, we can contrive an alternative explanation based on the trial order. For the sake of illustration, assume that the true success rate due to the perceptual factors is 0.55 for heat-seeking, 0.45 for random, and 0.4 for mixed conditions. That is, the difference in the success rate in trials with mixed movements to pure movements is not so dramatic. These values could be recovered if the different conditions were presented in blocks. Because the conditions are presented randomly, subjects need to use a single one-fits-them-all template when looking for a positive evidence in the display. Consider a strategy that focuses on heat-seeking and random movement. Let's say, it achieves 0.55 and 0.45 success with pure movement, but only 0.2 with mixed movement. The average success rate is $(0.55 + 0.45 + 4 \cdot 0.2)/6 = 0.3$. Now consider a strategy that focuses on the mixed conditions and achieves only 0.34 with heat-seeking and random movement, but only 0.2 in the non-mixed conditions. The average success rate is 0.29. Hence, the former strategy is preferred $(0.3 > 0.29)$. In conclusion, due to the randomization, the performance in the tasks in **Gao and Scholl** (2011) is potentially confounded by domain-general decision processes. This is, because decisions need to collapse evidence across trials with different stimulus properties.

REFERENCES

Gao, T. and Scholl, B. (2011), Chasing vs. stalking: Interrupting the perception of animacy., *Journal of Experimental Psychology: Human Perception and Performance*, 37, 3, 669–684

[1] The presented values are only approximate. They are used here for illustration of an argument.

2 SUPPLEMENTARY TABLES

Table 1. Data Overview

Block	Agent	Task	d	n_s	n_v/n_t	Label
1	Bug	LMA	0	13	42/42	D1.1.1
		LR	0	13	33.7/42	D1.1.2
2	Circle	LMA	$\{-0.15, -0.05, 0.05, 0.1, 0.15, 0.2\}$	5	42/42	D1.2.1
			$\{0.05, 0.1, 0.15, 0.2, 0.25, 0.3\}$	8	42/42	D1.2.2
		LR	$\{-0.15, -0.05, 0.05, 0.1, 0.15, 0.2\}$	5	39.2/42	D1.2.3
			$\{0.05, 0.1, 0.15, 0.2, 0.25, 0.3\}$	8	33.8/42	D1.2.4
3	Bug	DB	0	13	18/18	D1.3.1
	Circle	DB	$\{-0.15, -0.05, 0.05, 0.1, 0.15, 0.2\}$	5	22/22	D1.3.2
			$\{0.05, 0.1, 0.15, 0.20.25, 0.3\}$	8	22/22	D1.3.3
	Bug	SR	-	8	4.9/5	D1.3.4
		SL	-	8	5.0/5	D1.3.5
	Circle	SL	-	8	3.0/3	D1.3.6
1	Dart	LMA	0	28	42/42	D2.1.1
		LR	0	28	30.6/42	D2.1.2
2	Dart	LMA	$\{-0.4, -0.2\}$	16	42/42	D2.2.1
			$\{0.2, 0.4)$	9	42/42	D2.2.2
		LR	$\{-0.4, -0.2\}$	16	36.5/42	D2.2.3
			$\{0.2, 0.4\}$	9	36.1/42	D2.2.4
3	Dart	DB	0	25	18/18	D2.3.1
			$\{-0.4, -0.2\}$	16	22/22	D2.3.2
			$\{0.2, 0.4\}$	9	22/22	D2.3.3
		SR	-	23	4.7/5	D2.3.4
		SL	-	23	7.8/8	D2.3.5

n_s - number of subjects
n_v/n_t - proportion of trials that entered the analysis, with n_v the number of valid trials and n_t gives the total number of trials
Wherever n_v varied across subjects, an average across subjects is provided
d - pivot displacement
LMA - Leave-Me-Alone task, LR - Location Recall, DB - Distance Bisection Task, SR - recall of the position of the static stimuli, SL - localization of the static stimuli

Table 2. Overview of Fitted Models

Observed Variables	Model Specification	Label
$\mathbf{h}_{t,i}$ gives the vector of displacement in the Location Recall task for subject i on trial t<br>$\phi_{i,t,k}$ angle giving the direction of factor k on trial t for subject i	$h^x_{t,i} \sim \mathcal{N}(\sum_k \alpha_{k,i} \cos \phi_{i,t,k}, \sigma_{h,i})$<br>$h^y_{t,i} \sim \mathcal{N}(\sum_k \alpha_{k,i} \sin \phi_{i,t,k}, \sigma_{h,i})$<br>$\alpha_{k,i} \sim \mathcal{N}(\mu_{\alpha,k}, \sigma_{\alpha,k})$	S1.1
$\mathbf{h}_{t,i}, \phi_{i,t,k}$ same as in S1.1<br>$d_{t,i}$ pivot displacement as explained in figure 2	$\alpha_{4,i,t} = \gamma_i + \beta_i d_{t,i}$<br>$\gamma_i \sim \mathcal{N}(\mu_{\alpha,4}, \sigma_{\alpha,4})$<br>$\beta_i \sim \mathcal{N}(\mu_\beta, \sigma_\beta)$<br>otherwise same as in S1.1	S1.2
$w_{t,i}$ proportion of time spent in wolfpack areas by subject i on trial t	$w_{t,i} \sim \mathcal{B}(\mu_i, \nu_i)$<br>$\mu_i \sim \mathcal{B}(\mu_\mu, \nu_\mu)$	S2.1
$w_{t,i}$ as in S2.1<br>$d_{t,i}$ as in S1.2	$w_{t,i} \sim \mathcal{B}(\mu_i + \beta_i d_{t,i}, \nu_i)$<br>$\mu_i \sim \mathcal{N}(\mu_\mu, \sigma_\mu)$<br>$\beta_i \sim \mathcal{N}(\mu_\beta, \sigma_\beta)$	S2.2
$x_{t,i}, y_{t,i}$ displacement on the x and y axis of figure 2 for subject i on trial t	$x_{t,i} \sim \mathcal{N}(\mu_{x,i}, \sigma_{x,i})$<br>$y_{t,i} \sim \mathcal{N}(\mu_{y,i}, \sigma_{y,i})$<br>$\mu_{x,i} \sim \mathcal{N}(\mu_{\mu,x}, \sigma_{\mu,x})$<br>$\mu_{y,i} \sim \mathcal{N}(\mu_{\mu,y}, \sigma_{\mu,y})$	S3.1
$x_{t,i}, y_{t,i}$ as in S3.1<br>$d_{t,i}$ pivot displacement as explained in figure 2	$x_{t,i} \sim \mathcal{N}(\mu_{x,i} + \beta_{x,i} d_{t,i}, \sigma_{x,i})$<br>$y_{t,i} \sim \mathcal{N}(\mu_{y,i} + \beta_{y,i} d_{t,i}, \sigma_{x,i})$<br>$\beta_{x,i} \sim \mathcal{N}(\mu_{\beta,x}, \sigma_{\beta,x})$<br>$\beta_{y,i} \sim \mathcal{N}(\mu_{\beta,y}, \sigma_{\beta,y})$<br>$\mu_{x,i}, \mu_{y,i}$ as in S3.1	S3.2

In general, greek letters are used for parameters and roman letters are used for observed variables.
$\mathcal{N}(\mu, \sigma)$ - Gaussian distribution parametrized by mean μ and standard deviation σ
$\mathcal{B}(\mu, \nu)$ - Beta distribution parametrized by mean proportion μ and sample size ν

Table 3. Overview of Reported Analyses

Analysis	Data	Model	File	Prompt Number	Revision
R1.1	D1.1.2	S1.1	E1B1.ipynb	In[8] In[9]	In[3]
R1.2	D2.1.2	S1.1	E2B1.ipynb	In[6]	In[8]
R1.3	D2.2.3-4	S1.1	E2B2.ipynb	In[23]	In[11]
R1.4	D2.1.2; D2.2.3-4	S1.2	E2B2.ipynb	In[27] In[28]	In[12] In[13]
R1.5	D1.2.3-4	S1.2	E1B2.ipynb	In[7] In[8]	In[15]
R2.1	D1.1.1	S2.1	E1B1.ipynb	In[20]	In[4] In[5]
R2.2	D2.1.1	S2.1	E2B1.ipynb	In[18]	In[9]
R2.3	D2.2.1-2	S2.1	E2B2.ipynb	In[3]	In[17]
R2.4	D2.1.1; D2.2.1-2	S2.2	E2B2.ipynb	In[7]	In[18] In[19]
R2.5	D1.2.1-2	S2.2	E1B2.ipynb	In[4]	In[14]
R3.1	D1.3.4-6; D2.3.4-5	-	E2B3.ipynb	In[2]	-
R3.2	D1.3.1	S3.1	E1B3.ipynb	In[9]	In[20]
R3.3	D2.3.1-3	S3.1	E2B3.ipynb	In[9]	In[21]
R3.4	D2.3.1-3	S3.2	E2B3.ipynb	In[12]	In[22]
R3.5	D1.3.2-3	S3.2	E1B3.ipynb	In[13]	In[23]
